# Interleukin-7 Links T Lymphocyte and Intestinal Epithelial Cell Homeostasis

**DOI:** 10.1371/journal.pone.0031939

**Published:** 2012-02-27

**Authors:** Shabnam Shalapour, Katrin Deiser, Anja A. Kühl, Rainer Glauben, Susanne M. Krug, André Fischer, Özen Sercan, Stephane Chappaz, Stefan Bereswill, Markus M. Heimesaat, Christoph Loddenkemper, Michael Fromm, Daniela Finke, Günter J. Hämmerling, Bernd Arnold, Britta Siegmund, Thomas Schüler

**Affiliations:** 1 Institute of Immunology, Charité-Universitätsmedizin Berlin, Campus Benjamin Franklin, Berlin, Germany; 2 Institute of Pathology/Research Center ImmunoSciences, Charité-Universitätsmedizin Berlin, Campus Benjamin Franklin, Berlin, Germany; 3 Department of Gastroenterology, Infectiology and Rheumatology, Charité-Universitätsmedizin Berlin, Campus Benjamin Franklin, Berlin, Germany; 4 Institute of Clinical Physiology, Charité-Universitätsmedizin Berlin, Campus Benjamin Franklin, Berlin, Germany; 5 Institute of Microbiology and Hygiene, Charité-Universitätsmedizin Berlin, Campus Benjamin Franklin, Berlin, Germany; 6 Department of Molecular Immunology, German Cancer Research Center, Im Neuenheimer Feld 280, Heidelberg, Germany; 7 Department of Biomedicine, Division of Developmental Immunology, University of Basel, Basel, Switzerland; University of Auvergne, France

## Abstract

Interleukin-7 (IL-7) is a major survival factor for mature T cells. Therefore, the degree of IL-7 availability determines the size of the peripheral T cell pool and regulates T cell homeostasis. Here we provide evidence that IL-7 also regulates the homeostasis of intestinal epithelial cells (IEC), colon function and the composition of the commensal microflora. In the colon of T cell-deficient, lymphopenic mice, IL-7-producing IEC accumulate. IEC hyperplasia can be blocked by IL-7-consuming T cells or the inactivation of the IL-7/IL-7R signaling pathway. However, the blockade of the IL-7/IL-7R signaling pathway renders T cell-deficient mice more sensitive to chemically-induced IEC damage and subsequent colitis. In summary, our data demonstrate that IL-7 promotes IEC hyperplasia under lymphopenic conditions. Under non-lymphopenic conditions, however, T cells consume IL-7 thereby limiting IEC expansion and survival. Hence, the degree of IL-7 availability regulates both, T cell and IEC homeostasis.

## Introduction

Interleukin-7 (IL-7) is a crucial survival factor for T cells and the competition for IL-7 is the major regulatory principle that stabilizes peripheral T cell homeostasis [1], [2]. T cells express the IL-7 receptor (IL-7R) and remove IL-7 from the system continuously [3]. As soon as IL-7 production and consumption reach the equilibrium, the size of the peripheral T cell pool becomes self-limiting [1], [2]. Consequently, the lack of IL-7-consuming T cells is associated with increased levels of serum IL-7 in lymphopenic humans and mice [4], [5].

Host survival depends on the tight regulation of IL-7 availability. For example, mice lacking IL-7 suffer from severe immunodeficiency [6]. In contrast, elevated levels of IL-7 promote spontaneous T cell activation [7] and T cell-mediated inflammation in the intestine and other organs [8]-[10]. Similarly, the overabundance of IL-7 under lymphopenic conditions contributes to the activation of adoptively transferred, naïve T lymphocytes, which undergo lymphopenia-induced proliferation (LIP), convert into effector/memory T cells and cause inflammation in the large intestine [11], [12]. Based on the aforementioned observations, the blockade of IL-7R signaling in pathogenic T cells is considered as a therapeutic option for the treatment of T cell-mediated autoimmunity [13], [14].

However, recent evidence suggests that the maintenance of immunological self-tolerance in the intestine is not only controlled by cytokine receptor signaling in immune cells. For example, cell autonomous cytokine receptor signals regulate intestinal epithelial cell (IEC) homeostasis and protect mice from immune-mediated colitis [15]-[19]. We have shown recently that IEC are the major source of IL-7 in the murine intestine [20]. However, it remained open whether and how IL-7 affects IEC homeostasis and intestinal physiology. Here we show that murine IEC express functional IL-7R and expand in response to IL-7 *in vivo*. Furthermore, we demonstrate that IEC accumulate in the colon of lymphopenic mice in an IL-7/IL-7R-dependent fashion correlating with decreased colitis induction. Importantly, IEC hyperplasia and protection from colitis are blocked, if naive CD8^+^ T cells consume IL-7 or IL-7R signaling is inactive in IEC. In summary, our data demonstrate that IL-7 regulates IEC homeostasis and intestinal integrity. Under lymphopenic conditions, elevated levels of IL-7 promote IEC hyperplasia and protect mice from colitis. In contrast, IL-7 consumption by T cells prevents IEC hyperplasia and facilitates colitis induction. Hence, an as yet unknown IL-7-dependent regulatory feedback loop links T cell and IEC homeostasis and controls intestinal integrity.

## Results

### Lymphopenia-associated IEC hyperplasia leads to the accumulation of IL-7^+^ cells

**Figure 1:**
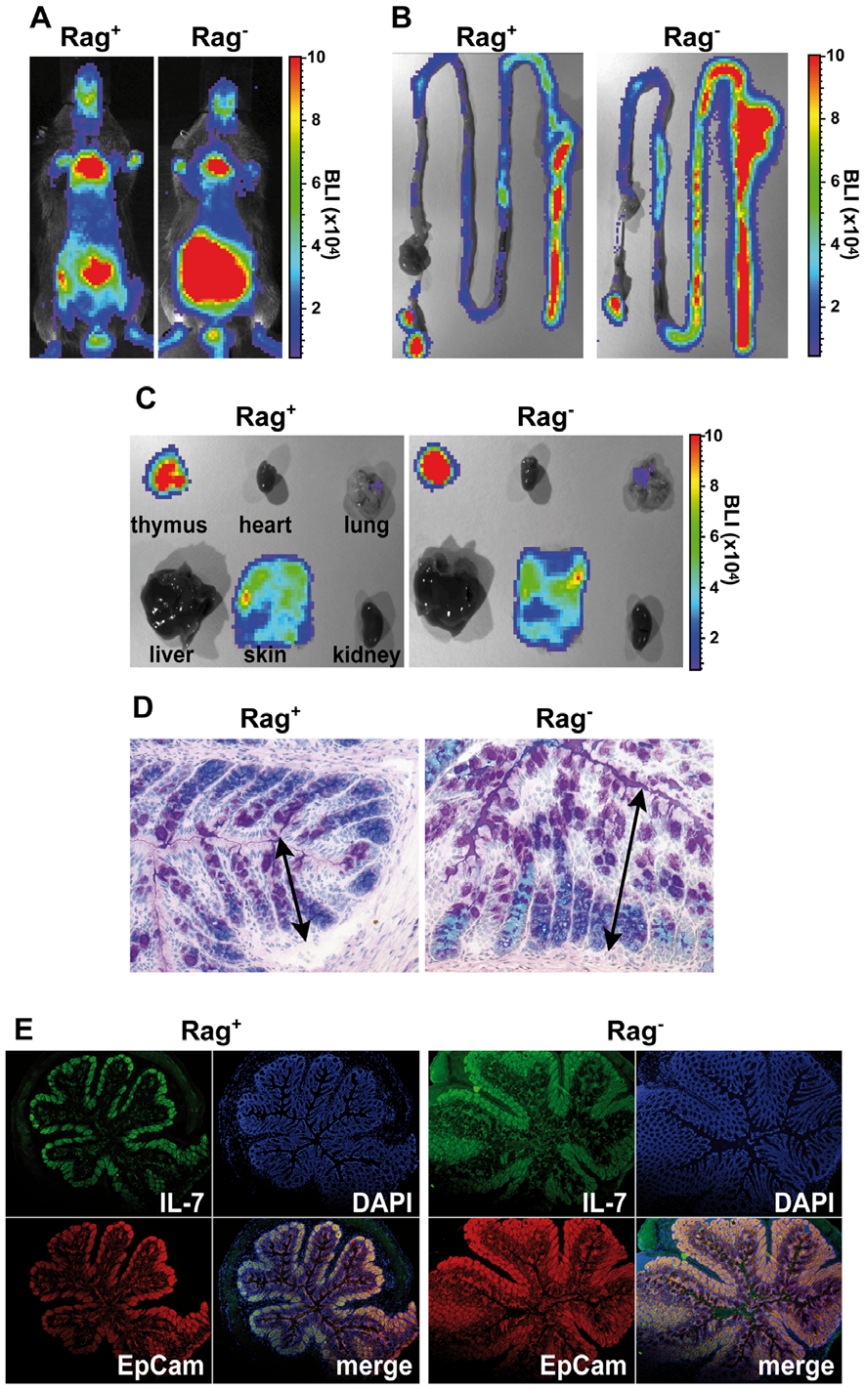
Elevated levels of IL-7 expression and IEC hyperplasia in the colon of Rag^−^ mice. (**A**) Representative bioluminescence (BL) images for Rag-competent (Rag^+^; n = 31) and Rag-deficient IL-7GCDL mice (Rag^−^; n = 21) are shown. BL was determined (**B**) in the intestine and (**C**) thymus, heart, lung, liver, skin and kidney of Rag^+^ (n = 12) and Rag^−^ IL-7GCDL mice (n = 12). (**A–C**) BL is shown in photons per s per cm^2^ per steradian. (**D, E**) Colon sections from Rag^+^ (n = 5–8) and Rag^−^ IL-7GCDL mice (n = 6–8) were stained with (**D**) periodic acid-Schiff (PAS)/Alcian blue (AB) or (**E**) DAPI and antibodies for IL-7 and EpCam. (**D**) Differentiated goblet cells stain positive for PAS (red) and appear purple/magenta. Acidic mucopolysaccharides/glycosaminoglycans are visualized by AB. Arrows indicate the distance between the basis of the crypts and the colon lumen. (**D, E**) Data are representative for 3 independent experiments and 2–3 staining reactions per mouse.

We have shown recently that the commensal microflora and Interferon-© (IFN-©) promote IL-7 production in the intestine [20]. However, it remained unclear whether T cells contribute to the regulation of intestinal IL-7 production. In order to visualize T cell-dependent *il-7* gene regulation *in vivo*, we made use of bacterial artificial chromosome (BAC)-transgenic IL-7-reporter mice. This mouse encodes enhanced green fluorescent protein (G), recombinase Cre (C), the human diphteria toxin receptor (D) and click beetle green luciferase 99 (L) under control of the *il-7* promoter and is termed IL-7GCDL hereafter. As we have shown previously, luciferase activity in IL-7GCDL mice correlates closely with *il-7* gene activity. IL-7GCDL mice were crossed to the Rag-deficient (Rag^−^) genetic background. As shown in Figure 1A, bioluminescence (BL) signals were most pronounced in the thorax and the abdomen of Rag^+^ and Rag^−^ IL-7GCDL mice. However, BL was strongly increased in the abdomen, but not the thorax, of Rag^−^ IL-7GCDL mice (Figure 1A). This resulted mainly from elevated levels of transgene expression in the intestine with particularly high levels in the colon (Figure 1B). In contrast, BL levels in thymus, skin and lung were similar in Rag^+^ and Rag^−^ IL-7GCDL mice (Figure 1C). Histological analysis revealed that the colon of Rag^−^ IL-7GCDL mice was hyperplastic (Figure 1D) and contained increased numbers of IL-7^+^ Epcam^+^ IEC (Figure 1E). Importantly, IEC hyperplasia was independent of the GCDL transgene and equally pronounced in non-transgenic Rag^−^ mice (Figure S1). Thus, lymphopenia-associated IEC hyperplasia is associated with the accumulation of IL-7^+^ IEC in the colon.

### Lymphopenia-associated IEC hyperplasia is IL-7/IL-7R-dependent

**Figure 2:**
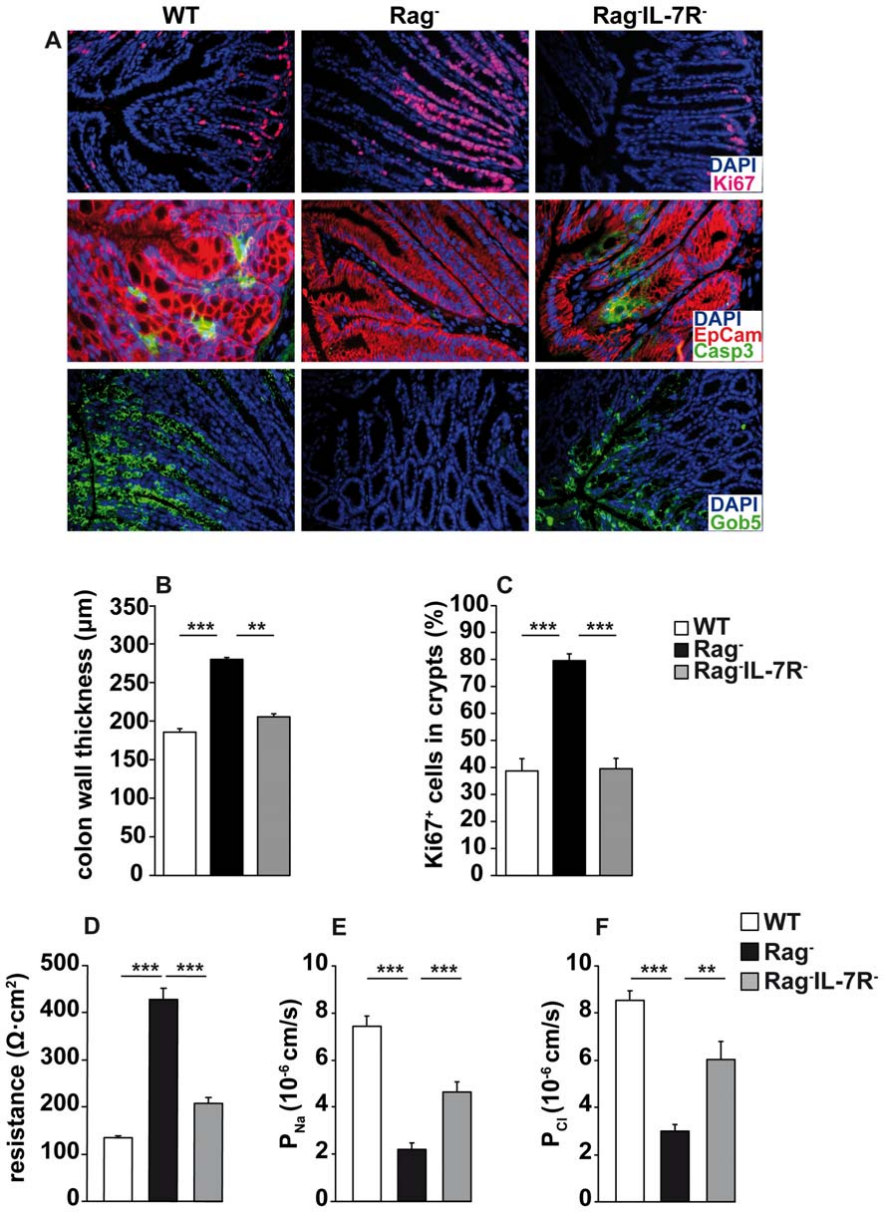
IL-7R signaling promotes lymphopenia-associated IEC hyperplasia and alters colon function. (**A**) Colon sections from WT (n = 13–16), Rag^−^ (n = 16–37) and Rag^−^IL-7R^−^ mice (n = 13) were stained with DAPI and antibodies for Ki67, EpCam, cleaved caspase 3 (Casp3) or Gob5. (**B**) Colon wall thickness (µm) and (**C**) the percentage of Ki67^+^ cells in crypts were determined for WT (n = 6), Rag^−^ (n = 12) and Rag^−^IL-7R^−^ (n = 6) mice. (**B, C**) 30–60 individual measurements were performed. (**A–C**) Data are representative for 4 independent experiments and 2–3 staining reactions per mouse. (**D**) Transepithelial resistance (Ω·cm^2^), and apparent permeabilities (P) for (**E**) Na^+^ and (**F**) Cl^−^ were determined for colon samples from WT (n = 6), Rag^−^ (n = 5) and Rag^−^IL-7R^−^ (n = 5) mice. Five to twelve independent measurements per experimental group were performed. (**B–F**) Shown are mean values+SEM. Statistically significant values are indicated: ** p<0.01 and *** p<0.001 (Student's t test).

IL-7 is a potent cell cycle-promoting and anti-apoptotic cytokine [21]. We therefore asked next, whether lymphopenia-associated IEC hyperplasia is IL-7-dependent. To test this, colon sections from IL-7GCDL non-transgenic WT, Rag^−^ and IL-7 receptor α chain (IL-7Rα)-deficient Rag^−^ (Rag^−^IL-7R^−^) mice were analyzed. As compared to WT mice, Rag^−^ crypts contained more Ki67^+^ proliferating cells (Figure 2A and C), were elongated (Figure 2A and B) and contained fewer cleaved caspase 3^+^ apoptotic cells (Figure 2A). Additionally, the number of IEC expressing Gob5 (mCLCA3) was strongly reduced in Rag^−^ mice (Figure 2A), indicating altered IEC differentiation. In contrast, colons of Rag^−^IL-7R^−^ did not show any signs of IEC hyperplasia. The frequency of Ki67^+^ cells (Figure 2A and C) and colon wall thickness (Figure 2A and B) were increased while numbers of cleaved caspase 3^+^ apoptotic and Gob5^+^ cells (Figure 2A) were reduced. Thus, IL-7R signaling promotes hyperplasia of IEC in the colon of Rag^−^ mice. A similar tendency was observed in the small intestine (Figure S2). However, these effects were far less pronounced than in the colon, probably due to lower levels of *il-7* expression (Figure 1B).

Next we asked whether IL-7R-dependent changes in IEC homeostasis were associated with alterations in gut physiology. For this purpose, transepithelial resistance (TER) and apparent permeabilities for Na^+^ and Cl^−^ were measured in the colon. As compared to T cell-competent WT mice, TER was elevated in Rag^−^ samples (Figure 2D), correlating well with the simultaneous reduction of Na^+^ and Cl^−^ permeability (Figure 2E, F). Compared to Rag^−^ samples, TER and permeabilities were restored again in Rag^−^IL-7R^−^ mice and nearly reached WT levels (Figure 2D–F). In effect, IL-7R-dependent IEC hyperplasia in Rag^−^ mice is associated with decreased intestinal permeability.

### IL-7 overabundance promotes IEC hyperplasia

**Figure 3:**
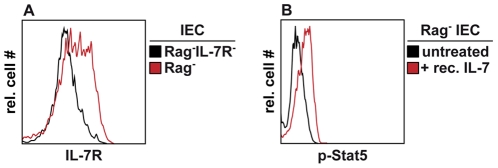
IEC express functional IL-7R. (**A, B**) IEC were isolated from the colon of (**A**) Rag^−^IL-7R^−^ and (**A, B**) Rag^−^ mice. (**B**) Rag^−^ IEC were stimulated with 20 ng/ml rec. IL-7 for 15 minutes or were left untreated. The levels of IL-7R expression and Stat5 phosphorylation (p-Stat5) were determined by flow cytometry. (**A, B**) Shown are relative cell numbers and relative fluorescence intensities for (**A**) IL-7R and (**B**) p-Stat5 after gating on viable (**A**) (7AAD^−^), (**A, B**) CD45^−^, EpCam^+^ IEC. Results are representative for 2–3 independent experiments.

**Figure 4:**
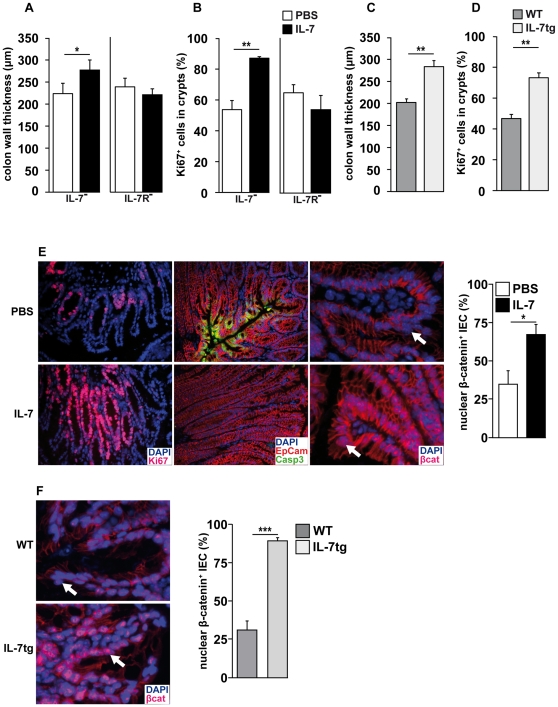
IL-7 promotes IEC proliferation and survival. (**A, B**) Rag^−^IL-7^−^ (IL-7^−^; n = 3) and Rag^−^IL-7R^−^ (IL-7R^−^; n = 3) mice were treated with PBS (white bars) or IL-7/anti-IL-7 (black bars) twice a week for 2 weeks. (**A, C**) Colon wall thickness (µm) and (**B, D**) the percentage of Ki67^+^ cells in crypts were determined in colon sections from (**A, B**) IL-7-treated Rag^−^IL-7^−^ and Rag^−^IL-7R^−^ mice and (**C, D**) untreated WT (n = 6) and IL-7tg (n = 5) mice. (**A**) 30–54, (**B**) 15–23, (**C**) 47–68 and (**D**) 29–32 individual measurements were performed per experimental group. Shown are mean values+SEM. Statistically significant values are indicated: * p<0.05 and ** p<0.01 (Student's t test). (**E**) Colon sections from PBS-treated (upper row) and IL-7/anti-IL-7-treated Rag^−^IL-7^−^ (lower row; n = 3) were stained with DAPI and antibodies for Ki67, EpCam, cleaved caspase 3 (Casp3) or β-catenin (βcat). (**F**) Colon sections from WT (n = 5) and IL-7tg mice (n = 6) were stained with DAPI and antibodies for β-catenin. (**E, F**) White arrows indicate nuclei. Bar diagrams show the percentage of luminal IEC with nuclear β-catenin. 130–280 nuclei per experimental group were counted. Shown are mean values+SEM. Statistically significant values are indicated: * p<0.05 and ** p<0.01 (Student's t test). (**A–F**) Data represent one experiment with a total of 23 individual mice and 2–3 independent staining reactions per mouse.

Freshly isolated colonic IEC from Rag^−^ mice expressed the IL-7R (Figure 3A) and phosphorylated signal transducer and activator of transcription (Stat) 5 after IL-7 treatment (Figure 3B). This suggests a direct action of IL-7 on IEC homeostasis. To test whether the restoration of IL-7 signaling is sufficient to induce IEC hyperplasia, IL-7-deficient Rag^−^ (Rag^−^IL-7^−^) mice were treated with recombinant mouse IL-7. To exclude IL-7R-independent side effects, Rag^−^IL-7R^−^ mice were treated in parallel. PBS-treated mice served as negative controls. As shown in Figure 4A and B, colon wall thickness and the numbers of Ki67^+^ cells/crypt were similar in the colons of PBS-treated Rag^−^IL-7^−^ and Rag^−^IL-7R^−^ mice. Hence, untreated Rag^−^IL-7^−^ mice, similar to Rag^−^IL-7R^−^ mice (Figure 2), did not show signs of IEC hyperplasia. In contrast, colons of IL-7-treated Rag^−^IL-7^−^ mice contained elevated numbers of IEC (Figure 4A and E) and showed a strong increase in IEC proliferation (Figure 4B and E). Additionally, the frequency of apoptotic cleaved caspase 3^+^ IEC was reduced (Figure 4E), indicating that IL-7 promotes both, IEC proliferation and survival. This resulted, at least partially, from a direct effect of IL-7 on IEC, which showed increased levels of nuclear Stat5 (Figure S3). These effects were IL-7R-dependent, since IEC homeostasis remained unaltered in IL-7-treated Rag^−^IL-7R^−^ mice (Figure 4A and B). These results indicate, that the reduction of IEC numbers in Rag^−^IL-7^−^ and Rag^−^IL-7R^−^ mice does not result from unknown developmental defects but from the lack of IL-7 signaling.

Due to the lack of IL-7-consuming T cells, IL-7 availability is increased in Rag^−^ mice [5]. This suggested to us that the intestinal epithelium responds to elevated IL-7 levels. To test this hypothesis, colon sections from IL-7 transgenic (tg) mice [22] were analyzed. As shown in Figure 4C, their colonic epithelium was hyperplastic and contained far more Ki67^+^ cells than that of non-transgenic WT mice (Figure 4D). Thus, IL-7 overabundance is sufficient to cause IEC hyperplasia.

IL-7 can induce nuclear translocation of β-catenin [23], a central regulator of IEC homeostasis [24]. In the nucleus, β-catenin binds to transcription factors of the T cell factor/lymphocyte enhancer factor (TCF/LEF) family to activate genes promoting proliferation, survival, differentiation and positioning of IEC [24]. In the WT colon, nuclear β-catenin is mainly restricted to the crypt base [24] (data not shown). Accordingly, luminal IEC of PBS-treated Rag^−^IL-7^−^ mice were nearly devoid of nuclear β-catenin (Figure 4E). In contrast, IL-7-treatment caused the accumulation of β-catenin in the nucleus of luminal IEC (Figure 4E). Similar results were obtained with IL-7tg mice (Figure 4F). Hence, IL-7 overabundance is associated with the accumulation of nuclear β-catenin in luminal IEC and IEC hyperplasia.

### T lymphocytes prevent IEC hyperplasia and promote colitis in an antigen-independent, IL-7R-dependent fashion

**Figure 5:**
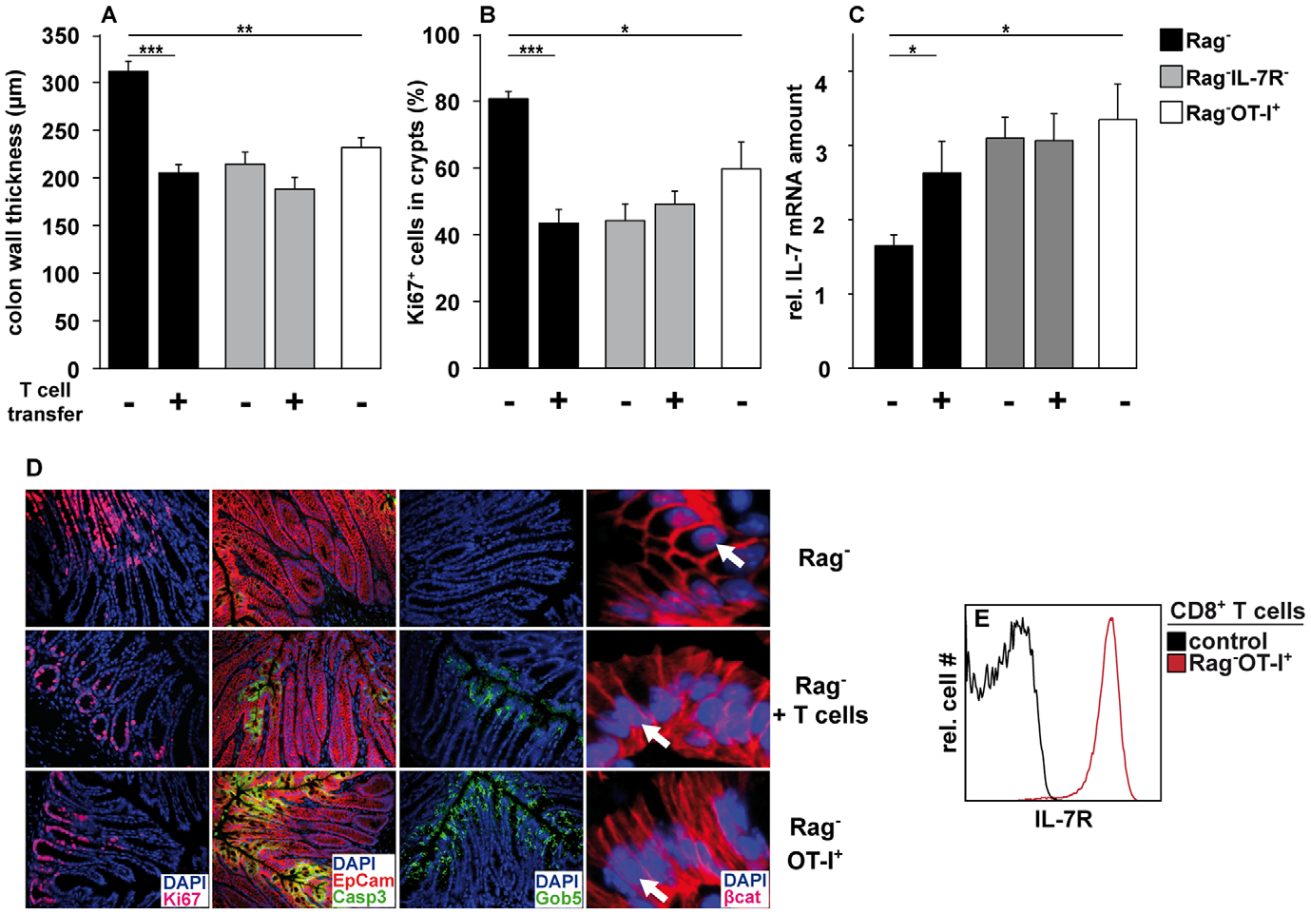
T lymphocytes regulate IEC homeostasis in an antigen-independent, IL-7R-dependent fashion. (**A, B**) Rag^−^ (n = 6) and Rag^−^IL-7R^−^ (n = 7) were reconstituted with 5×10^6^ polyclonal CD4^+^ and CD8^+^ T cells from Thy1.1-congenic mice. Untreated Rag^−^ (n = 8) and Rag^−^IL-7R^−^ (n = 7) served as controls. Eighty-five days after transfer, colon sections were prepared and analyzed for (**A**) colon wall thickness (µm) and (**B**) the percentage of Ki67^+^ cells in crypts. Data are representative for 2 independent experiments. (**A, B**) Colon sections from untreated Rag^−^OT-I^+^ mice (n = 5) were analyzed in parallel. (**A**) 45–62 and (**B**) 26–43 individual measurements were performed per experimental group. (**C**) Relative IL-7 mRNA levels were determined in relation to β-actin mRNA levels in the colon of the indicated groups (Rag^−^ n = 12; Rag^−^+T cells n = 8; Rag^−^ IL-7R^−^ n = 8; Rag^−^ IL-7R^−^+T cells n = 8; Rag^−^ OTI^+^ n = 5). (**A–C**) Shown are mean values+SEM. Statistically significant values are indicated: * p<0.05, ** p<0.01, *** p<0.001 (Student's t test). (**D**) Colon sections from untreated Rag^−^ (n = 37), T cell-reconstituted Rag^−^ (n = 6) and Rag^−^OT-I^+^ mice (n = 5) were stained with DAPI and antibodies for Ki67, EpCam, cleaved caspase 3 (Casp3), Gob5 or β-catenin (βcat). White arrows indicate nuclei. Data are representative for at least two independent experiments and 2–3 staining reactions per mouse. (**E**) Rag^−^OT-I^+^ spleen cells were analyzed by flow cytometry. Shown are relative cell numbers and fluorescence intensities for IL-7R on CD8^+^ OT-I T cells (Rag^−^OT-I^+^). In control samples, primary antibodies were omitted. Results are representative for 2 independent experiments.

Having shown that lymphopenia-associated IL-7 overabundance promotes IEC hyperplasia, we asked next whether IL-7 consumption by T cells is sufficient to normalize IEC homeostasis in Rag^−^ mice. For this purpose, Rag^−^ mice were reconstituted with polyclonal CD4^+^ and CD8^+^ T lymphocytes and colon sections were analyzed 85 days later. As compared to untreated Rag^−^ controls, colon wall thickness (Figure 5A) and the number of Ki67^+^ cells (Figure 5B and D) were reduced in T cell-reconstituted Rag^−^ mice. Simultaneously, the number of apoptotic cleaved caspase 3^+^ and Gob5^+^ cells (Figure 5D) was elevated after T cell reconstitution. Additionally, nuclear β-catenin was hardly detectable in luminal IEC of T cell-reconstituted Rag^−^ mice (Figure 5D). In agreement with reduced IEC numbers and a normalization of IEC homeostasis, BL was strongly reduced in the intestine of T cell-reconstituted Rag^−^IL-7GCDL mice (Figure S4). In contrast, T cell reconstitution did not lead to any overt changes in the colonic epithelium of Rag^−^IL-7R^−^ control mice (Figure 5A and B). Similarly, IEC homeostasis remained unaltered in Rag^−^ mice reconstituted with IL-7R^−^ T cells (Figure S5). However, at this stage we could not exclude that antigen-recognition and activation of the transferred T cells, in conjunction with IL-7R signaling, caused the subsequent regulation of IEC homeostasis. To test this possibility, the colon of Rag^−^ OT-I^+^ TCR-transgenic (Rag^−^OT-I^+^) mice was analyzed. These mice are devoid of T and B lymphocytes except for a monoclonal population of CD8^+^ T cells, which are specific for chicken ovalbumin and therefore inert to foreign antigens. Importantly, CD8^+^ T cells from untreated Rag^−^OT-I^+^ mice did not show any signs of activation and had a naïve phenotype (Figure S7). As shown in Figure 5A, colon wall thickness in Rag^−^OT-I^+^ mice was significantly lower than in Rag^−^ mice. Furthermore, fewer proliferating Ki67^+^ (Figure 5B and D) and more apoptotic cleaved caspase 3^+^ IEC were found in Rag^−^OT-I^+^ mice (Figure 5D). At the same time, high numbers of Gob5^+^ cells were found in Rag^−^OT-I^+^ colons and luminal IEC contained hardly any nuclear β-catenin (Figure 5D). Thus, IEC homeostasis in Rag^−^OT-I^+^ mice was very similar to WT (Figure 2A–C) and T cell-reconstituted Rag^−^ mice (Figure 5A, B and D). We therefore conclude that naïve CD8^+^ T cells can regulate IEC homeostasis in an antigen-independent fashion. However, T cell-mediated IEC regulation requires IL-7R expression on both cells types, T cells (Figure S5) and IEC (Figure 5A–C). It is important to stress that CD8^+^ OT-I T cells expressed much higher levels of the IL-7Rα chain (Figure 5E) than IEC (Figure 3A). This suggests that naïve CD8^+^ OT-I T cells consume more IL-7 than IEC. If so, the presence or absence of T cells should affect IL-7R-dependent gene expression in IEC. In this context it was shown recently, that IL-7R signaling counter-regulates *il-7* gene activity in a negative feedback loop [5]. Consequently, IL-7 overabundance in Rag^−^ mice is associated with decreased rates of *il-7* transcription in lymph node stroma cells [5]. As shown in Figure 5C, IL-7 mRNA levels in the colon of untreated Rag^−^ mice were significantly lower than in untreated Rag^−^IL-7R^−^ mice. This indicates that IL-7 overabundance in Rag^−^ mice reduces *il-7* gene expression in IEC similar to lymph node stroma cells [5]. After T cell reconstitution, however, IL-7 mRNA levels increased in Rag^−^ mice and reached levels similar to those found in untreated Rag^−^IL-7R^−^ mice. Importantly, T cell reconstitution did not alter IL-7 mRNA levels in the colon of Rag^−^IL-7R^−^ mice.

In Rag^−^OT-I^+^ mice, IL-7 mRNA levels in the colon were significantly higher than in untreated Rag^−^ mice but comparable to T cell-reconstituted Rag^−^ mice. Hence, the presence of naïve CD8^+^ T cells is sufficient to increase *il7* expression in IL-7R-competent IEC. This suggests that T cells withdraw IL-7 from IEC thereby regulating IL-7R-dependent gene expression and subsequent IEC homeostasis.

**Figure 6:**
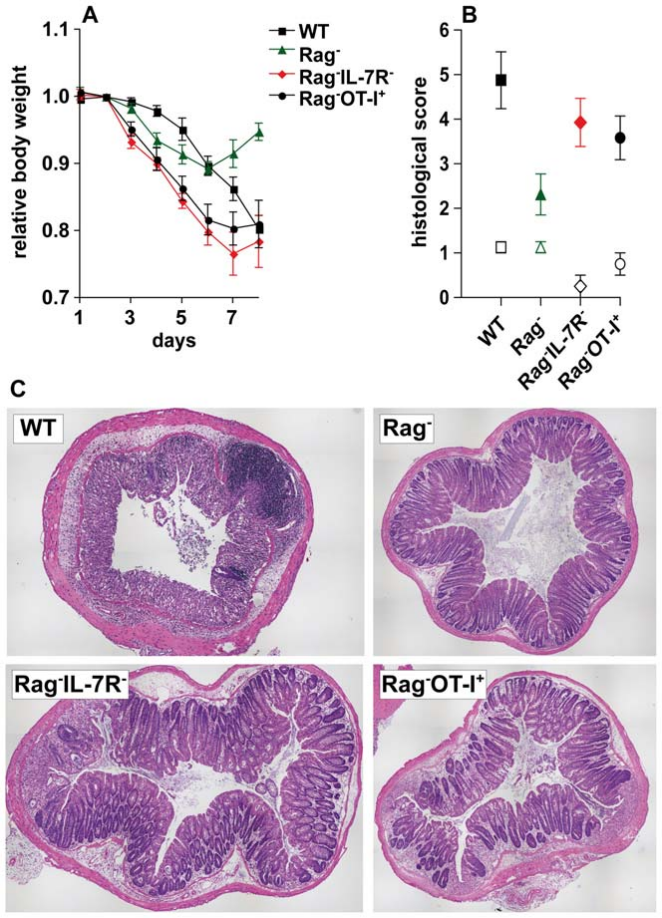
IL-7R signaling protects Rag^−^ mice from DSS-induced colitis. (**A, B**) WT (n = 4), Rag^−^ (n = 8), Rag^−^IL-7R^−^ (n = 7) and Rag^−^OT-I^+^ mice (n = 6) received dextran sulfate sodium (DSS) via the drinking water. From day 5 on, DSS-free drinking water was provided. (**A**) Body weight was determined every day and calculated in relation to the initial body weight. Shown are the mean relative body weight ± SEM and the time after onset of DSS treatment. (**B, C**) Colon samples were taken at day 8 and analyzed histologically. Shown are histological scores for groups of untreated (open symbols; n = 4) and DSS-treated mice (closed symbols; n = 6–8). (**C**) Shown are representative colon sections from the indicated mice. (**A–C**) Data represent one experiment.

To test for the physiological relevance of our findings, we made use of a well-defined disease model. Mice were treated for 5 days with dextran sulfate sodium (DSS) via the drinking water to induce IEC damage and subsequent colitis. As shown in Figure 6A, WT mice lost weight rapidly without any apparent signs of recovery after DSS withdrawal at day 5. In contrast, body weight loss was only modest and transient in Rag^−^ mice (Figure 6A). However, DSS-resistance of Rag^−^ mice was IL-7R-dependent as shown by the fact that Rag^−^IL-7R^−^ mice lost around 20–25% of their body weight and hardly recovered (Figure 6A).

DSS-induced colitis is more severe in WT than in Rag^−^ mice, because IEC damage facilitates priming of commensal-specific, pro-inflammatory T cells [25], which aggravate disease. However, based on the aforementioned results we hypothesized that T cells may promote DSS-induced colitis in an antigen-independent fashion, namely via the blockade of IL-7R signaling in IEC. To test this, Rag^−^OT-I^+^ mice were treated with DSS. The kinetics of body weight loss was nearly identical to Rag^−^IL-7R^−^ mice (Figure 6A). Importantly, CD8^+^ T cells from DSS-treated Rag^−^OT-I^+^ mice did not show any signs of activation and had a naïve phenotype (Figure S7). Hence, the presence of naive, IL-7-consuming CD8^+^ T cells is sufficient to block the protective effect of IL-7 in DSS-treated Rag^−^ mice.

As expected, tissue damage and leukocyte infiltration were most pronounced in WT mice (Figure 6B and C). In contrast, Rag^−^ colon samples showed only mild alterations as compared to untreated controls. However, tissue damage and leukocyte infiltration were clearly more pronounced in Rag^−^IL-7R^−^ and Rag^−^OT-I^+^ mice as compared to DSS-treated Rag^−^ mice (Figure 6B and C). In summary, our results demonstrate that IL-7 protects Rag^−^ mice from DSS-induced tissue damage and subsequent colitis.

## Discussion

CD4^+^ T cells recognizing antigens derived from the commensal microflora cause colitis under lymphopenic conditions [26]. Since IL-7 is a potent activation, growth and survival factor for T cells, it promotes intestinal damage via its action on T cells [9], [11], [12], [27]. Here we provide evidence that IL-7 regulates IEC homeostasis in the colon and protects lymphopenic mice from DSS-induced colitis.

We show that IL-7-producing IEC accumulate in the intestine of Rag^−^ mice (Figure 1). This requires IL-7R signaling, correlates with increased rates of IEC proliferation and survival and results in IEC hyperplasia (Figure 2A–C). IEC are the major source of IL-7 in the colon [20] and express IL-7R, which are functional *in vitro* (Figure 3) and *in vivo* (Figure S3). This suggests that IL-7R signaling in IEC contributes to the regulation of intestinal homeostasis. Nevertheless, we cannot formally exclude indirect effects of IL-7, e.g. on innate lymphoid cells (ILC). IL-22-producing ILC are important regulators of intestinal homeostasis [28], they are generated in an IL-7/IL-7R-dependent fashion [29], [30] and protect Rag^−^ mice from DSS-induced colitis [31], [32]. Therefore, the lack of ILC may promote alterations in IEC homeostasis and aggravate DSS-induced colitis in Rag^−^IL-7R^−^ mice (Figure 2 and 6).

Nevertheless, the lack of ILC does not explain the results we have obtained with IL-7R-competent Rag^−^ mice. For example, only Rag^−^ mice reconstituted with IL-7R-competent (Figure 5) but not -deficient T cells normalized IEC homeostasis (Figure 5 and S5A–C) although ILC were similarly abundant in both experimental groups (Figure S5D). Furthermore, IL-7R-competent Rag^−^OT-I^+^ mice contained ILC (Figure S5E) but nevertheless showed reduced rates of IEC proliferation/survival (Figure 5A–D) and more severe symptoms after DSS treatment (Figure 6). Finally, IEC numbers and proliferation were indistinguishable between lethally irradiated Rag^−^ mice reconstituted with Rag^−^ or Rag^−^IL-7R^−^ bone marrow (BM) (Information S1 and Figure S6). This shows that lymphopenia-associated IEC hyperplasia can occur independently of BM-derived IL-7R^+^ ILC and might result form IL-7R signaling in IEC.

Hence, our results indicate that IL-7R expression by naïve T cells is sufficient to limit IEC proliferation/survival and aggravate DSS colitis even in the presence of ILC. Together with the fact that the T cell-mediated normalization of IEC homeostasis required IL-7R expression by both, T and host cells (Figure S5A–C and Figure 5A and B), our data suggest that T cells are sufficient to regulate intestinal homeostasis via the withdrawal of IL-7 from the intestinal epithelium. Nevertheless, we do not want to exclude that T cells may produce an as yet unknown, IL-7-induced factor, that also contributes to the regulation of IEC homeostasis.

T cell reconstitution was associated with two seemingly contradictory observations in the intestine of Rag^−^ mice. On the one hand it increased *il-7* gene activity in IEC (Fig. 5C), on the other, reporter gene activity was decreased in the intestine of Rag^−^ IL-7GCDL mice (Figure S4D). It is important to emphasize that the bioluminescent signal of an organ is determined by both, the transcriptional activity of the *il-7* gene and the total number of IL-7-producing cells per organ. Since IEC numbers were strongly reduced in the intestine of T cell-reconstituted Rag^−^ mice (Figure 5A, B, D and Figure S4A–C) the comparably weak upregulation of *il-7* gene activity (Figure 5D) could not prevent the overall reduction of intestinal IL-7 production (Figure S4D). Hence, T cells appear to regulate steady state IL-7 levels in the intestine of Rag^−^ mice mainly via the modulation of IEC numbers.

It is important to note that IEC homeostasis in Rag^−^IL-7R^−^ mice was similar to WT mice (Figure 2A–C). This shows that IL-7R signaling is dispensable for IEC proliferation and survival in the steady state. Nevertheless, IL-7 overabundance promotes IL-7R signaling in a considerable proportion of IEC (Figure S3) correlating with elevated levels of IEC proliferation and survival (Figure 4). We therefore conclude that lymphopenia-related IL-7 overabundance [5] is the major reason for IEC hyperplasia in Rag^−^ mice (Figures 2 and 4) and protection from DSS-induced colitis (Figure 6). Whether the protective effect of IL-7 is due to increased rates of (i) steady state IEC proliferation/survival or (ii) more efficient tissue regeneration in Rag^−^ mice is an important question for the future.

In previous experiments we have analyzed the colon of IL-7GCDL mice after crossing them to reporter mice expressing eGFP only after the Cre-mediated deletion of a DNA stop cassette [20]. Since the IL-7GCDL mouse expresses Cre (but not eGFP) under control of the *il-7* promoter, this approach enabled us to visualize IL-7 producing IEC and their progeny based on eGFP expression. In the colon of such F1 animals, entire crypts were eGFP positive [20] suggesting that the *il-7* promoter is active in epithelial stem cells. This interpretation is supported by our current findings shown in Figure 1E and S1. Here, IL-7 expression is most prominent at the crypt base where stem cells are located [24]. Hence, colonic epithelial stem cells are a putative source of IL-7. However, it remains to be shown which other IEC subtypes express IL-7 and its receptor to elucidate whether IL-7 regulates IEC homeostasis in lymphopenic mice in an autocrine and/or paracrine fashion.

We have shown previously that the commensal microflora promotes *il-7* gene expression in the intestine [20]. Consequently, intestinal BL and corresponding IL-7 levels in the colon (data not shown) were strongly reduced in antibiotic-treated Rag^−^IL-7GCDL mice (Figure S8A). This was associated with reduced colon wall thickness (Figure S8B) and lower levels of IEC proliferation (Figure S8C). Similar results were obtained with germ-free Rag^−^ mice (Figure S8B and C) showing that the commensal microflora promotes IEC hyperplasia in Rag^−^ mice, probably via the induction of *il-7* gene expression [20].

It is known that the bacterial content of the large intestine is higher than in the small intestine. This may explain why *il-7* gene activity is comparably low in the latter (Figure 1B) and why IEC homeostasis in this part of the gut is less severely affected by IL-7 (Figure S2). However, IL-7-dependent hyperplasia of the colonic epithelium correlates with changes in colon function (Figure 2D–F) and alterations in the commensal microflora (Information S1 and Figure S8).

IL-7 levels are elevated in HIV patients [4], which frequently suffer from diarrhea [33]. Similarly, severe combined immunodeficiency (SCID) patients often develop intestinal complications [34]. Due the broad impact of IL-7 on intestinal physiology shown here, it is tempting to speculate that the lymphopenia-associated overabundance of IL-7 promotes intestinal alterations frequently observed in lymphopenic patients.

The nuclear translocation of β-catenin is crucial for the maintenance of IEC homeostasis [24]. In a healthy colon, nuclear β-catenin is mainly restricted to IEC located at the crypt base [24]. However, in the colon of Rag^−^ mice, nuclear β-catenin was also found in luminal IEC (Figure 5D). This was not the case for Rag^−^IL-7^−^ (Figure 4E) and Rag^−^IL-7R^−^ mice (data not shown) suggesting that IL-7R signaling promotes nuclear translocation of β-catenin in Rag^−^ IEC, similar to what was described for myeloma cells [23]. In line with this, IL-7 injection and its transgenic overexpression were both sufficient to cause the accumulation of nuclear β-catenin in IEC (Figure 4E, F). In the nucleus, β-catenin initiates a transcriptional program that promotes cell cycle progression [35], [36] and survival [37]. This may facilitate wound healing after DSS-induced IEC damage and may explain why Rag^−^ mice were protected from colitis in an IL-7R-dependent fashion (Figure 6).

IEC hyperplasia is an early step in colon carcinogenesis [38] and facilitates malignant transformation in the mouse intestine [18], [39]. Hence, IL-7/IL-7R-dependent IEC hyperplasia in Rag^−^ mice may be a double-edged sword. While it improves resistance to chemically-induced IEC damage, it may increase the risk for colon cancer development at the same time.

Due to their high levels of IL-7R expression (Figure 5E), T lymphocytes are major IL-7 consumers in the body. It is well accepted that T lymphocytes compete for IL-7 and that the degree of IL-7 availability determines the size of the peripheral T cell pool [1], [2]. Here we provide evidence that the degree of IL-7 availability also determines the size of the IEC pool and that competition for IL-7 is not restricted to T cells. As we have shown here, IL-7R^+^ T cells alter β-catenin expression, IEC homeostasis and *il-7* gene expression in Rag^−^ but not Rag^−^IL-7R^−^ mice (Figure 5). This process seems to be independent of TCR specificity, since naïve ovalbumin-specific CD8^+^ OT-I T cells normalized the indicated parameters similarly efficient like polyclonal T lymphocytes (Figure 5). Based on these results, we propose that T lymphocytes and IEC homeostasis are linked via the competition for IL-7. In the presence of an intact T cell pool, IL-7 levels are too low to affect IEC homeostasis. Under lymphopenic conditions, however, IL-7 overabundance promotes IEC hyperplasia and protects the epithelium from tissue damage (Figure 6). Hence, the immune status determines whether and how IL-7 affects intestinal homeostasis.

In summary, our results provide new insights into the regulation of intestinal homeostasis and have important implications for the design of clinical protocols targeting the IL-7/IL-7R signaling pathway to treat T cell-mediated autoimmunity and cancer [13], [14], [40].

## Materials and Methods

### Ethics Statement

This study was performed in strict accordance with the recommendations for the Care and Use of Laboratory Animals at the Charité-Universitätsmedizin Berlin. The protocol was approved by the Landesamt für Gesundheit und Soziales-Berlin (Permit Number: G0170/08). Every effort was made to minimize suffering.

### Mice

C57BL/6J, Thy1.1-congenic (B6.PL-Thy1a/Cy), IL-7GCDL [20], Rag1-deficient (Rag^−^) (B6.129S7-Rag1^tm1Mom^/J), IL-7^−^ and IL-7R^−^ mice (B6.129S7-Il7r^tm1Imx^/J) on the Rag^−^ background (Rag^−^IL-7^−^ and Rag^−^IL-7R^−^, respectively), IL-7 transgenic (IL-7tg) [22] and Rag1-deficient OT-I (Rag^−^OT-I^+^) (all on a C57BL/6 background) were bred under specific pathogen-free conditions in our animal facilities. Germ-free Rag^−^ mice were kindly provided by M. Dorsch (Hannover Medical School, Hannover, Germany). All animal experiments were performed according to institutional guidelines.

### Adoptive T cell transfer

CD4^+^ and CD8^+^ T cells were isolated from single cell suspensions prepared from spleens and lymph nodes of Thy1.1-congenic WT mice using CD4- and CD8α-specific microbeads and autoMACS (both Miltenyi Biotec GmbH). 5×10^6^ cells were injected i.v.

### Bioluminescence (BL) detection

D-luciferin (Synchem OHG) and the IVIS Imaging Systems (either Series 100 or 200) (Xenogen) were used. For BL detection in live animals, 3 mg D-luciferin were injected i.p. into shaved mice. For BL detection in isolated organs, mice were injected i.v. with 3 mg D-luciferin 2 minutes prior to organ removal. Organs were collected in phosphate-buffered saline (PBS) containing 1.5 mg/ml D-luciferin and analyzed subsequently.

### Immunostaining

Immunostainings were done as described previously [20]. To determine colon wall thickness, the distance between submucosa and lumen was measured. Six to eight areas were analyzed per mouse and the mean colon wall thickness was calculated. The percentage of Ki67^+^ cells/crypt was determined for 5 to 6 crypts per mouse and mean values were calculated.

### Antibodies

Ab specific for the following mouse antigens were used: mAb rat anti-EpCam (G8.8; kindly provided by G. Moldenhauer, DKFZ, Heidelberg, Germany), pAb goat anti-IL-7 (R&D Systems), mAb rabbit anti-cleaved Caspase 3 (Cell Signaling Technology), mAb rat anti-Ki67 (TEC-3; Dako), mAb rabbit anti-β-catenin (6B3; Cell Signaling Technology), mAb pStat5 (y694, 47; BD Biosciences) and pAb rabbit anti-Gob5 (Abcam). The following Alexa 594-, Alexa 647-, Alexa 488-conjugated secondary antibodies were used: donkey anti-rat IgG, donkey anti-rabbit IgG, donkey anti-goat IgG, goat anti-rat IgG (Molecular Probes).

### Transepithelial resistance and permeability measurements

Colon samples were rinsed with Ringer's solution, glued on plastic rings (serosal area of the tissue without correction: 0.049 cm^2^) with histoacryl tissue glue and mounted in Ussing chambers for measuring transepithelial resistance (TER, Ω ·cm^2^) and dilution potentials. Resistance of the bathing solution was measured prior to the experiment and subtracted. Ussing hemichambers and water-jacketed gas lifts were filled with 10 ml standard Ringer's solution (in mM: Na^+^ 140; Cl^−^ 149.8; K^+^ 5.4; Ca^2+^ 1.2; Mg^2+^ 1; HEPES 10; D(+)-glucose 10. pH was adjusted to 7.4 with NaOH. The solution was equilibrated with 100% O_2_ at 37°C. NaCl dilution potentials were measured by switching one hemichamber to a solution containing a reduced concentration of NaCl and all other components identical to standard Ringer's. Osmolality was balanced by mannitol. Apparent ion permeabilities for sodium and chloride were determined from dilution potentials using the Goldman-Hodgkin-Katz equation. For relating TER and permeabilities to the effective epithelial area, the area of crypt epithelium was determined. For this, colon samples were glued to plastic rings as used in the Ussing chamber. Crypt number per mm^2^ (n_crypt_/A_serosa_) was determined by counting crypts of Clarke's reagent fixed tissue under a light microscope. To evaluate the crypt length (L_crypt_) and inner and outer crypt diameter (ID_crypt_; OD_crypt_), cross sections of tissues were fixed with 10% formalin, embedded in paraffin, HE-stained, and analyzed. From the evaluated parameters area correction factors were calculated by equation 1:
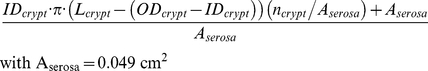
(1)


### Quantification of IL-7 mRNA

IL-7 mRNA was quantified as described previously [20].

### IL-7 injection

A complex of 5 µg of recombinant mouse IL-7 (eBioscience) and 50 µg anti-mouse IL-7 (M25) in PBS was injected i.p. 2×/week for 2 weeks as described before [41].

### Isolation of colonic IEC and flow cytometric analysis of IL-7R expression

Freshly isolated colons were incised longitudinally, washed in PBS/EDTA (2 mM), and subsequently incubated for 30 min at 37°C in RPMI1640 supplemented with 10% FCS, Penicillin/Streptomycin, 0.2 mg/ml Collagenase D, 0.2 mg/ml Dispase II and 10 µg/ml Dnase I (Roche Diagnostics). After incubation, epithelial cells were detached with the help of a syringe plunger and free crypts were incubated in PBS/EDTA (5 mM) for 10 min at 4°C. Dissociated cells were passed through a 40 µm cell strainer (BD Biosciences) and washed in PBS/EDTA (5 mM). Cells were stained with directly labeled monoclonal antibodies against CD45 (30F-11, BD Biosciences), CD326 (G8.8, Biolegend) and CD127 (A7R34, eBioscience) for 45 min at 4°C. Two rounds of signal amplification were performed with the anti-APC FASER kit (Miltenyi Biotec GmbH) to increase the fluorescence intensity for CD127. Data were acquired on a Canto II flow cytometer (BD Biosciences) and analyzed with FlowJo software (Tree Star).

### Colitis induction and histopathological analysis

Acute colitis was induced by feeding mice with 4% dextran sulfate sodium (DSS, MW: 36000–50000, MP Biomedicals) dissolved in drinking water and administered ad libitum for 5 days followed by 3 days of regular drinking water, resulting in a 8-day experimental period. For histological analysis, colon samples were fixed with 4% paraformaldehyde and embedded in paraffin. 2 µm sections were cut, deparaffinized, stained with hematoxylin and eosin (H&E), and scored in a blinded manner. The DSS-induced histological colitis score is the sum of individual scores for inflammatory cell infiltration and tissue damage. Individual scores for infiltration were given as follows: 0 points, no inflammatory cell infiltration; 1 point, increased number of inflammatory cells in lamina propria; 2 points, inflammatory cell infiltration extends into the submucosa; 3 points, transmural inflammatory infiltrates; and for tissue damage: 0 points, no mucosal damage; 1 points, discrete epithelial lesions; 2 points, erosions or focal ulcerations; 3 points, severe mucosal damage with extended ulcerations extending into bowel wall.

### Statistics

Data are given as means+SEM and tested for significance by Student's t-test or the Mann-Whitney U-test, as appropriate. In cases of multiple testing, the Bonferroni-Holm correction was applied. P<0.05 was considered significant.

## Supporting Information

Figure S1 **IEC hyperplasia in non-transgenic Rag^−^ mice is associated with the accumulation of IL-7^+^ IEC.** Colon sections from WT (n = 5) and Rag^−^ mice (n = 6) were stained with DAPI and antibodies for IL-7 and EpCam. Data are representative for 2 independent experiments and 2–3 staining reactions per mouse.
(TIF)

Figure S2 **IEC homeostasis in the small intestine is only slightly affected by IL-7R signaling.** (**A, B**) Tissue sections from the small intestine (SI) of WT (n = 4), Rag^−^ (n = 5) and Rag^−^IL-7R^−^ mice (n = 4) were stained with DAPI and antibodies for Ki67, EpCam or cleaved caspase 3 (Casp3). (**A**) SI wall thickness (µm) is shown. Data are representative for 44–48 individual measurements per experimental group. Shown are mean values+SEM. Statistically significant values are indicated (*; Student's t test).
(TIF)

Figure S3 **IL-7R signaling induces nuclear accumulation of Stat5 in colonic IEC.** Rag^−^IL-7^−^ (n = 3) and Rag^−^IL-7R^−^ (n = 3) mice were treated with PBS or IL-7/anti-IL-7 (IL-7) twice a week for 2 weeks as described in Figure 4. Colon sections were stained with DAPI and antibodies for Stat5 (green) and EpCam (red). The white arrow indicates a nucleus containing Stat5. Data represent are representative for 2 independent staining reactions per mouse.
(TIF)

Figure S4 **IEC homeostasis and IL-7 reporter activity are normalized in T cell-reconstituted Rag^−^ IL-7GCDL mice.** (**A–C**) Rag^−^ IL-7GCDL mice (Rag^−^) were reconstituted with 5×10^6^ MACS-sorted CD4^+^ and CD8^+^ T lymphocytes isolated from spleens and lymph nodes of Rag^+^ IL-7GCDL mice (Rag^+^). Colon sections were analyzed 56 days later. Colon sections from untreated Rag^+^ IL-7GCDL mice (Rag^+^), untreated Rag^−^ IL-7GCDL mice (Rag^−^) and T cell-reconstituted Rag^−^ IL-7GCDL mice (Rag^−^+T cells) were stained with DAPI and antibodies for (**A**) Ki67, (**B**) Cleaved-caspase 3 or (**C**) β-catenin. Results are representative for 4–7 mice and up to 10 independent staining reactions per group. (**D**) Shown are representative BL measurements from the intestine of untreated Rag^−^ IL-7GCDL mice (Rag^−^; n = 3) and T cell-reconstituted Rag^−^ IL-7GCDL mice (Rag^−^+T cells; n = 3) 56 days after T cell transfer. BL is shown in photons per s per cm^2^ per steradian. Data represent one experiment.
(TIF)

Figure S5 **IL-7R-deficient T cells do not regulate IEC homeostasis.** (**A–D**) Rag^−^ mice were reconstituted with 1×10^6^ MACS-sorted CD4^+^ and CD8^+^ T lymphocytes isolated from spleens and lymph nodes of IL-7R^−^ mice (Rag^−^+T^IL-7R-^; n = 3). Controls were left untreated (Rag^−^; n = 4). Colon sections were stained with DAPI and (**A–C**) antibodies for Ki67, EpCam, Cleaved-caspase 3 (Casp3) or β-catenin (βcat) 100 days after transfer. Data are representative for 2 independent staining reactions per mouse. Shown are (**A**) colon wall thickness (µm) and (**B**) the percentage of Ki67^+^ cells in crypts as mean values+SEM. Values were not significantly different (n.s.: p>0.05; Student's t test). (**D**) Colon sections from Rag^−^ mice reconstituted with WT T lymphocytes (Rag^−^+T^WT^; see Figure 5) or IL-7R^−^ T cells (Rag^−^+T^IL-7R-^) or (**E**) untreated Rag^−^OT-I^+^ mice were stained with DAPI and antibodies for IL-7R and CD3 to visualize transferred T cells (CD3^+^IL-7R^+/−^) and innate lymphoid cells (CD3^−^IL-7R^+^). Results are representative for 2 independent staining reactions per mouse.
(TIF)

Figure S6 **IL-7R expression on non-hematopoietic cells is sufficient for IEC hyperplasia in the colon of Rag^−^ mice.** (**A–C**) Lethally irradiated Rag^−^ mice were reconstituted with bone marrow cells from Rag^−^ mice (Rag^−^→Rag^−^; n = 4) or Rag^−^IL-7R^−^ (Rag^−^IL-7R^−^→Rag^−^; n = 3). (**A**) Colon wall thickness (µm; 46–56 individual measurements per group) and (**B**) the percentage of Ki67^+^ cells in crypts (at least 500 nuclei per group) were determined 9 weeks later. (**A, B**) Shown are mean values+SD. Statistically significant values are indicated: n.s., not significant; * p<0.05 and ** p<0.01 (Student's t test). (**D**) Colon sections from Rag^−^→Rag^−^ (n = 4) and Rag^−^IL-7R^−^→Rag^−^ chimeras (n = 3) were stained with DAPI and antibodies for Ki67. Upper row: 100× magnification; lower row: 400× magnification. Data are representative 2–3 staining reactions per mouse.
(TIF)

Figure S7 **DSS treatment does not activate CD8^+^ OT-I T cells.** CD8^+^ OT-I T cells were isolated from the spleen and mesenteric lymph node (MLN) of untreated (untr.; n = 8) and DSS-treated (DSS; n = 6; see Figure 6) Rag^−^OT-I^+^ mice. Activated CD8^+^ OT-I T cells (act.) were recovered from spleens of Rag^−^ mice (n = 10) 21–25 days after adoptive transfer and homeostatic expansion of 1×10^6^ cells. (**A**) The percentage of naive CD44^lo^CD62L^hi^ OT-I T cells was determined by flow cytometry after gating on CD8^+^Thy1.1^+^ cells. (**B**) CD8^+^ OT-I cells from the indicated sources were stimulated with their cognate peptide SIINFEKL for 6 hours and the percentage of Interferon-© (IFN-©)-positive cells was determined by flow cytometry after gating on CD8^+^Thy1.1^+^ cells. Data are representative for 1 (DSS) and 2 (act.) experiment(s).
(TIF)

Figure S8 **The commensal microflora promotes intestinal IL-7 production, which induces IEC hyperplasia and alterations in the commensal microflora.** (A) Rag^−^IL-7GCDL mice (n = 6) were treated with antibiotics for 49 days as described previously [20]. The same mouse prior to (untr) and after antibiotic treatment (anti) is shown. BL is shown in photons per s per cm^2^ per steradian. Similar results were obtained after antibiotic treatment for 21 days in 3 additional experiments (n = 21). (B) Colon wall thickness (µm) and (C) the percentage of Ki67^+^ cells in crypts were determined in colon sections from untreated (untr; n = 3) and antibiotic-treated Rag^−^IL-7GCDL mice (anti; n = 6) as well as specific-pathogen-free (spf; n = 3) and germ-free Rag^−^ mice (gf; n = 10). 5–7 individual crypts per mouse were analyzed. Shown are mean values+SEM. Statistically significant values are indicated: * p<0.05 and *** p<0.001 (Student's t test). (B, C) For antibiotic-treated mice, crypt length and the percentage of Ki67^+^ cells in crypts were analyzed for the representative experiment shown in (A). For germ-free mice, pooled data from two independent experiments are shown. Data are representative for 2–3 independent staining reactions per mouse. (D) Feces samples from WT (n = 18), Rag^−^ (n = 18) and Rag^−^IL-7R^−^ (n = 12) mice were analyzed by qRT-PCR to determine the copy number of eubacterial 16 S rDNA (EUBV3) and 16 S rRNA from *Lactobacillus-*group (LACTO), *Mouse Intestinal Bacteroides* (MIB), *Bacteroides/Prevotella-group* (BACT), gamma Proteobacteria/Enterobacteriaceae (ENTERO), *Clostridium leptum* subgroup (CLEP), *Clostridium coccoides* subgroup and *Enterococcus* (ECCOC). Shown are 16 S copy numbers per ng DNA (log10) for individual mice and the median for each experimental group. Statistically significant values are indicated: * p<0.05, ** p<0.01, *** p<0.001 (Mann-Whitney-U-Test).
(TIF)

Information S1 **Experimental procedures for the generation of bone marrow chimeras (Figure S6) and the analysis of the intestinal microflora (Figure S8) are described.** Specific references are included.
(DOCX)

## References

[1] Jameson SC (2002). Maintaining the norm: T-cell homeostasis.. Nat Rev Immunol.

[2] Khaled AR, Durum SK (2002). Lymphocide: cytokines and the control of lymphoid homeostasis.. Nat Rev Immunol.

[3] Park JH, Yu Q, Erman B, Appelbaum JS, Montoya-Durango D (2004). Suppression of IL7Ralpha transcription by IL-7 and other prosurvival cytokines: a novel mechanism for maximizing IL-7-dependent T cell survival.. Immunity.

[4] Napolitano LA, Grant RM, Deeks SG, Schmidt D, De Rosa SC (2001). Increased production of IL-7 accompanies HIV-1-mediated T-cell depletion: implications for T-cell homeostasis.. Nat Med.

[5] Guimond M, Veenstra RG, Grindler DJ, Zhang H, Cui Y (2009). Interleukin 7 signaling in dendritic cells regulates the homeostatic proliferation and niche size of CD4+ T cells.. Nat Immunol.

[6] von Freeden-Jeffry U, Vieira P, Lucian LA, McNeil T, Burdach SE (1995). Lymphopenia in interleukin (IL)-7 gene-deleted mice identifies IL-7 as a nonredundant cytokine.. J Exp Med.

[7] Kieper WC, Tan JT, Bondi-Boyd B, Gapin L, Sprent J (2002). Overexpression of interleukin (IL)-7 leads to IL-15-independent generation of memory phenotype CD8+ T cells.. J Exp Med.

[8] Sawa S, Kamimura D, Jin GH, Morikawa H, Kamon H (2006). Autoimmune arthritis associated with mutated interleukin (IL)-6 receptor gp130 is driven by STAT3/IL-7-dependent homeostatic proliferation of CD4+ T cells.. J Exp Med.

[9] Watanabe M, Ueno Y, Yajima T, Okamoto S, Hayashi T (1998). Interleukin 7 transgenic mice develop chronic colitis with decreased interleukin 7 protein accumulation in the colonic mucosa.. J Exp Med.

[10] Weitzmann MN, Roggia C, Toraldo G, Weitzmann L, Pacifici R (2002). Increased production of IL-7 uncouples bone formation from bone resorption during estrogen deficiency.. J Clin Invest.

[11] Totsuka T, Kanai T, Nemoto Y, Makita S, Okamoto R (2007). IL-7 Is essential for the development and the persistence of chronic colitis.. J Immunol.

[12] Tomita T, Kanai T, Nemoto Y, Totsuka T, Okamoto R (2008). Systemic, but Not Intestinal, IL-7 Is Essential for the Persistence of Chronic Colitis.. J Immunol.

[13] Watanabe M, Yamazaki M, Okamoto R, Ohoka S, Araki A (2003). Therapeutic approaches to chronic intestinal inflammation by specific targeting of mucosal IL-7/IL-7R signal pathway.. Curr Drug Targets Inflamm Allergy.

[14] Ponchel F, Cuthbert RJ, Goëb V (2011). IL-7 and lymphopenia.. Clin Chim Acta.

[15] Bollrath J, Phesse TJ, von Burstin VA, Putoczki T, Bennecke M (2009). gp130-mediated Stat3 activation in enterocytes regulates cell survival and cell-cycle progression during colitis-associated tumorigenesis.. Cancer Cell.

[16] Grivennikov S, Karin E, Terzic J, Mucida D, Yu GY (2009). IL-6 and stat3 are required for survival of intestinal epithelial cells and development of colitis-associated cancer.. Cancer Cell.

[17] Nenci A, Becker C, Wullaert A, Gareus R, van Loo G (2007). Epithelial NEMO links innate immunity to chronic intestinal inflammation.. Nature.

[18] Xiao H, Gulen MF, Qin J, Yao J, Bulek K (2007). The Toll-interleukin-1 receptor member SIGIRR regulates colonic epithelial homeostasis, inflammation, and tumorigenesis.. Immunity.

[19] Zaph C, Troy AE, Taylor BC, Berman-Booty LD, Guild KJ (2007). Epithelial-cell-intrinsic IKK-beta expression regulates intestinal immune homeostasis.. Nature.

[20] Shalapour S, Deiser K, Sercan O, Tuckermann J, Minnich K (2010). Commensal microflora and interferon-gamma promote steady-state interleukin-7 production in vivo.. Eur J Immunol.

[21] Jiang Q, Li WQ, Aiello FB, Mazzucchelli R, Asefa B (2005). Cell biology of IL-7, a key lymphotrophin.. Cytokine Growth Factor Rev.

[22] Mertsching E, Burdet C, Ceredig R (1995). IL-7 transgenic mice: analysis of the role of IL-7 in the differentiation of thymocytes in vivo and in vitro.. Int Immunol.

[23] Li Y, Chen W, Ren J, Yu WH, Li Q (2003). DF3/MUC1 signaling in multiple myeloma cells is regulated by interleukin-7.. Cancer Biol Ther.

[24] van der Flier LG, Clevers H (2009). Stem cells, self-renewal, and differentiation in the intestinal epithelium.. Annu Rev Physiol.

[25] Izcue A, Coombes JL, Powrie F (2009). Regulatory lymphocytes and intestinal inflammation.. Annu Rev Immunol.

[26] Feng T, Wang L, Schoeb TR, Elson CO, Cong Y (2010). Microbiota innate stimulation is a prerequisite for T cell spontaneous proliferation and induction of experimental colitis.. J Exp Med.

[27] Yamazaki M, Yajima T, Tanabe M, Fukui K, Okada E (2003). Mucosal T cells expressing high levels of IL-7 receptor are potential targets for treatment of chronic colitis.. J Immunol.

[28] Sonnenberg GF, Fouser LA, Artis D (2011). Border patrol: regulation of immunity, inflammation and tissue homeostasis at barrier surfaces by IL-22.. Nat Immunol.

[29] Satoh-Takayama N, Lesjean-Pottier S, Vieira P, Sawa S, Eberl G (2010). IL-7 and IL-15 independently program the differentiation of intestinal CD3-NKp46+ cell subsets from Id2-dependent precursors.. J Exp Med.

[30] Vonarbourg C, Mortha A, Bui VL, Hernandez PP, Kiss EA (2010). Regulated expression of nuclear receptor RORγt confers distinct functional fates to NK cell receptor-expressing RORγt(+) innate lymphocytes.. Immunity.

[31] Zenewicz LA, Yancopoulos GD, Valenzuela DM, Murphy AJ, Stevens S (2008). Innate and adaptive interleukin-22 protects mice from inflammatory bowel disease.. Immunity.

[32] Sawa S, Lochner M, Satoh-Takayama N, Dulauroy S, Bérard M (2011). RORγt+ innate lymphoid cells regulate intestinal homeostasis by integrating negative signals from the symbiotic microbiota.. Nat Immunol.

[33] Wilcox CM, Saag MS (2008). Gastrointestinal complications of HIV infection: changing priorities in the HAART era.. Gut.

[34] Buckley RH (2004). Molecular defects in human severe combined immunodeficiency and approaches to immune reconstitution.. Annu Rev Immunol.

[35] Tetsu O, McCormick F (1999). Beta-catenin regulates expression of cyclin D1 in colon carcinoma cells.. Nature.

[36] He TC, Sparks AB, Rago C, Hermeking H, Zawel L (1998). Identification of c-MYC as a target of the APC pathway.. Science.

[37] Zhang Z, Hartmann H, Do VM, Abramowski D, Sturchler-Pierrat C (1998). Destabilization of beta-catenin by mutations in presenilin-1 potentiates neuronal apoptosis.. Nature.

[38] Kinzler KW, Vogelstein B (1996). Lessons from hereditary colorectal cancer.. Cell.

[39] Guasch G, Schober M, Pasolli HA, Conn EB, Polak L (2007). Loss of TGFbeta signaling destabilizes homeostasis and promotes squamous cell carcinomas in stratified epithelia.. Cancer Cell.

[40] Sportès C, Gress RE, Mackall CL (2009). Perspective on potential clinical applications of recombinant human interleukin-7.. Ann N Y Acad Sci.

[41] Boyman O, Ramsey C, Kim DM, Sprent J, Surh CD (2008). IL-7/anti-IL-7 mAb complexes restore T cell development and induce homeostatic T Cell expansion without lymphopenia.. J Immunol.

