# Supplementary figures and images for: Interleukin-7 Links T Lymphocyte and Intestinal Epithelial Cell Homeostasis
Source: PLoS One. 2012 Feb 27;7(2):e31939. doi: 10.1371/journal.pone.0031939 (PMC3288069; doi:10.1371/journal.pone.0031939)

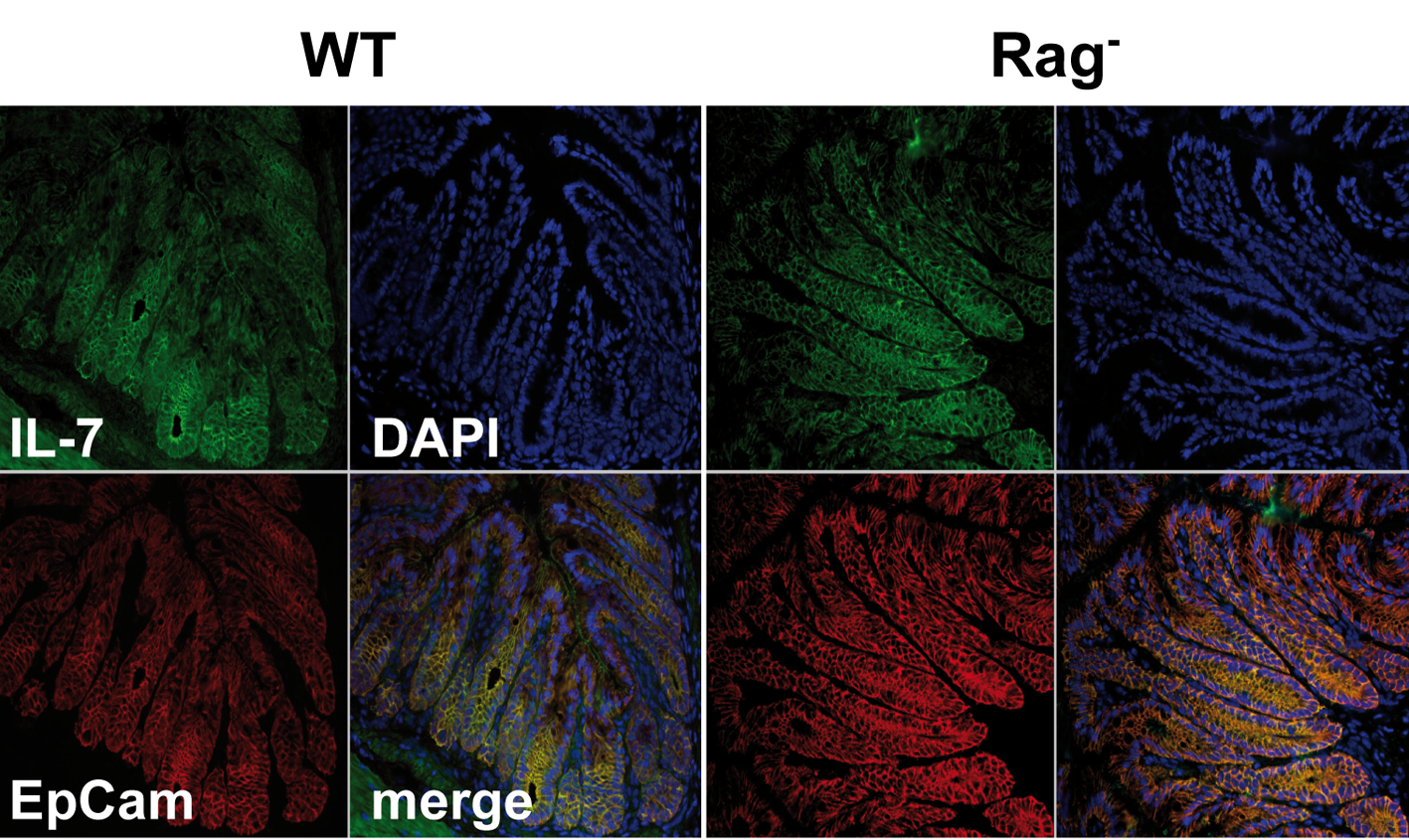

Supplement: Figure S1 — IEC hyperplasia in non-transgenic Rag− mice is associated with the accumulation of IL-7+ IEC. Colon sections from WT (n = 5) and Rag− mice (n = 6) were stained with DAPI and antibodies for IL-7 and EpCam. Data are representative for 2 independent experiments and 2–3 staining reactions per mouse. (TIF) [file pone.0031939.s001.tif]

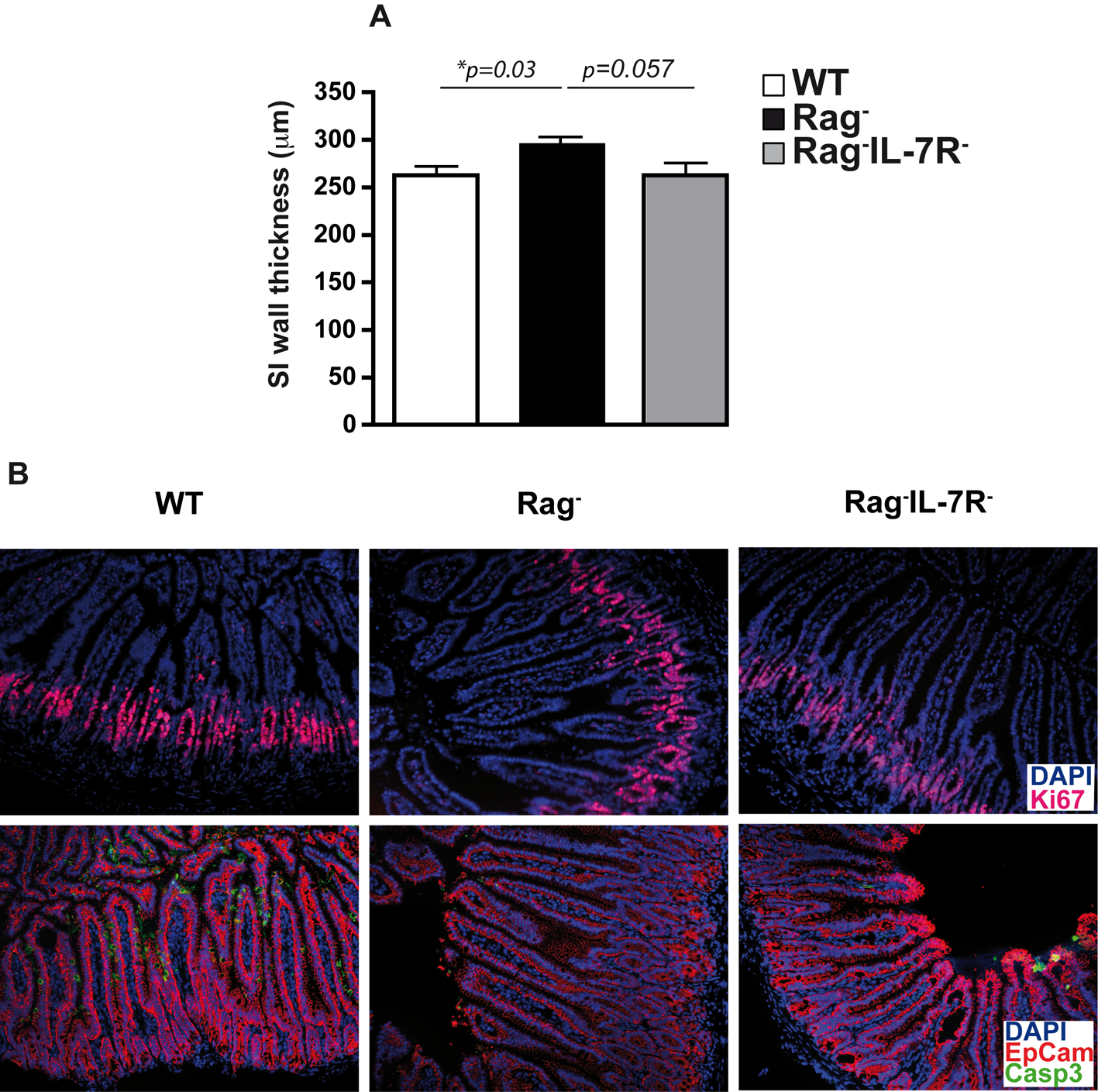

Supplement: Figure S2 — IEC homeostasis in the small intestine is only slightly affected by IL-7R signaling. (A, B) Tissue sections from the small intestine (SI) of WT (n = 4), Rag− (n = 5) and Rag−IL-7R− mice (n = 4) were stained with DAPI and antibodies for Ki67, EpCam or cleaved caspase 3 (Casp3). (A) SI wall thickness (µm) is shown. Data are representative for 44–48 individual measurements per experimental group. Shown are mean values+SEM. Statistically significant values are indicated (*; Student's t test). (TIF) [file pone.0031939.s002.tif]

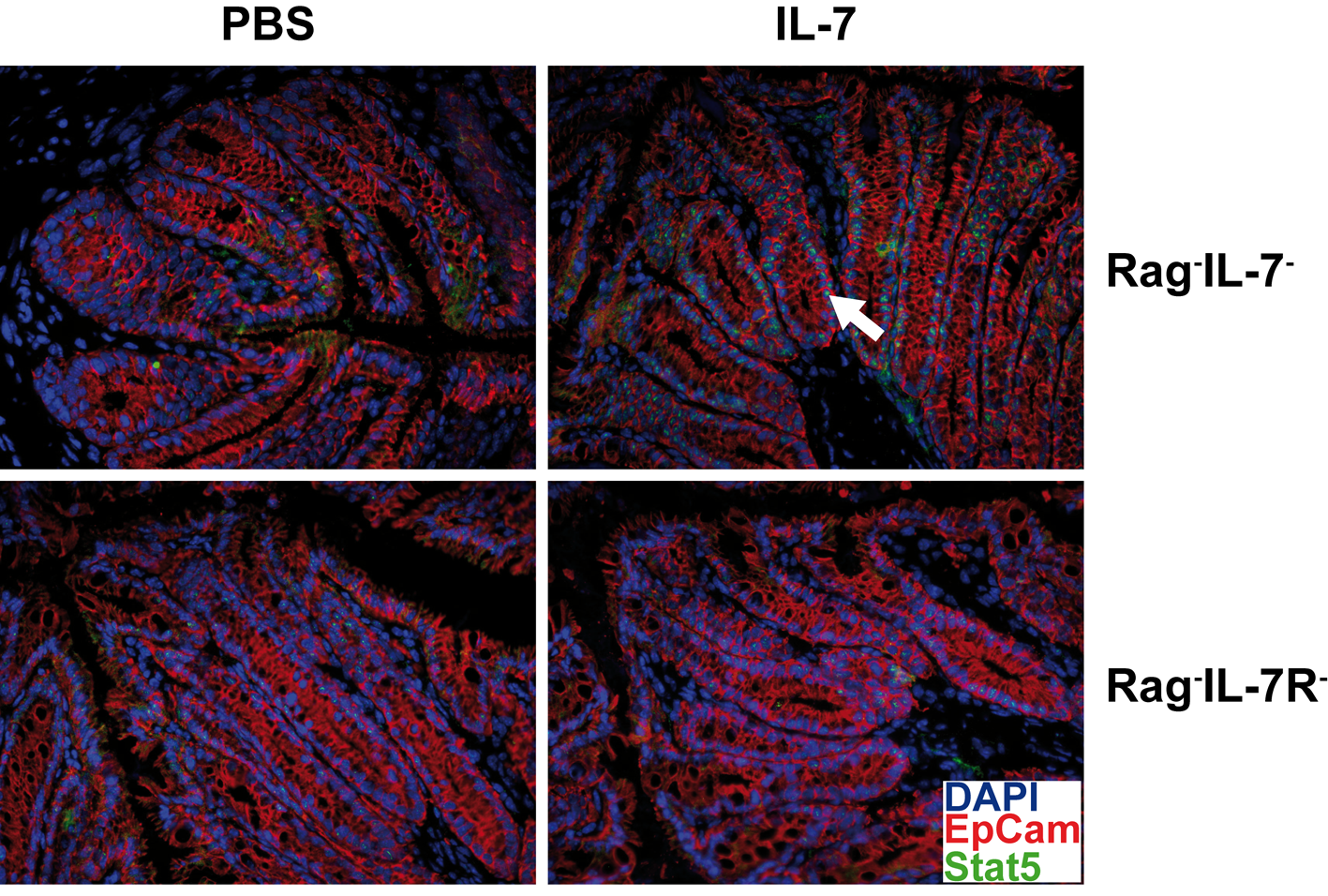

Supplement: Figure S3 — IL-7R signaling induces nuclear accumulation of Stat5 in colonic IEC. Rag−IL-7− (n = 3) and Rag−IL-7R− (n = 3) mice were treated with PBS or IL-7/anti-IL-7 (IL-7) twice a week for 2 weeks as described in Figure 4. Colon sections were stained with DAPI and antibodies for Stat5 (green) and EpCam (red). The white arrow indicates a nucleus containing Stat5. Data represent are representative for 2 independent staining reactions per mouse. (TIF) [file pone.0031939.s003.tif]

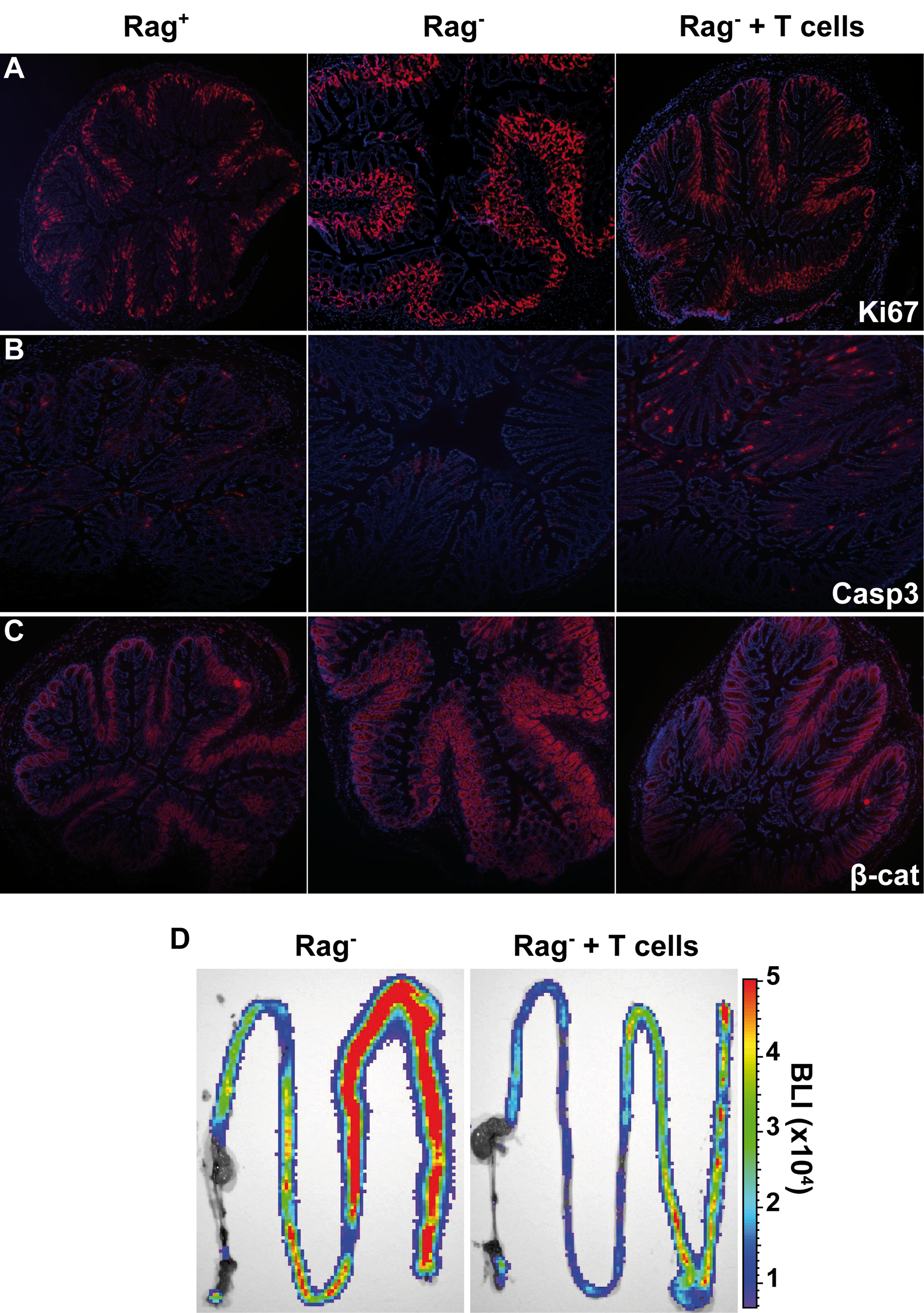

Supplement: Figure S4 — IEC homeostasis and IL-7 reporter activity are normalized in T cell-reconstituted Rag− IL-7GCDL mice. (A–C) Rag− IL-7GCDL mice (Rag−) were reconstituted with 5×10^6^ MACS-sorted CD4+ and CD8+ T lymphocytes isolated from spleens and lymph nodes of Rag+ IL-7GCDL mice (Rag+). Colon sections were analyzed 56 days later. Colon sections from untreated Rag+ IL-7GCDL mice (Rag+), untreated Rag− IL-7GCDL mice (Rag−) and T cell-reconstituted Rag− IL-7GCDL mice (Rag−+T cells) were stained with DAPI and antibodies for (A) Ki67, (B) Cleaved-caspase 3 or (C) β-catenin. Results are representative for 4–7 mice and up to 10 independent staining reactions per group. (D) Shown are representative BL measurements from the intestine of untreated Rag− IL-7GCDL mice (Rag−; n = 3) and T cell-reconstituted Rag− IL-7GCDL mice (Rag−+T cells; n = 3) 56 days after T cell transfer. BL is shown in photons per s per cm2 per steradian. Data represent one experiment. (TIF) [file pone.0031939.s004.tif]

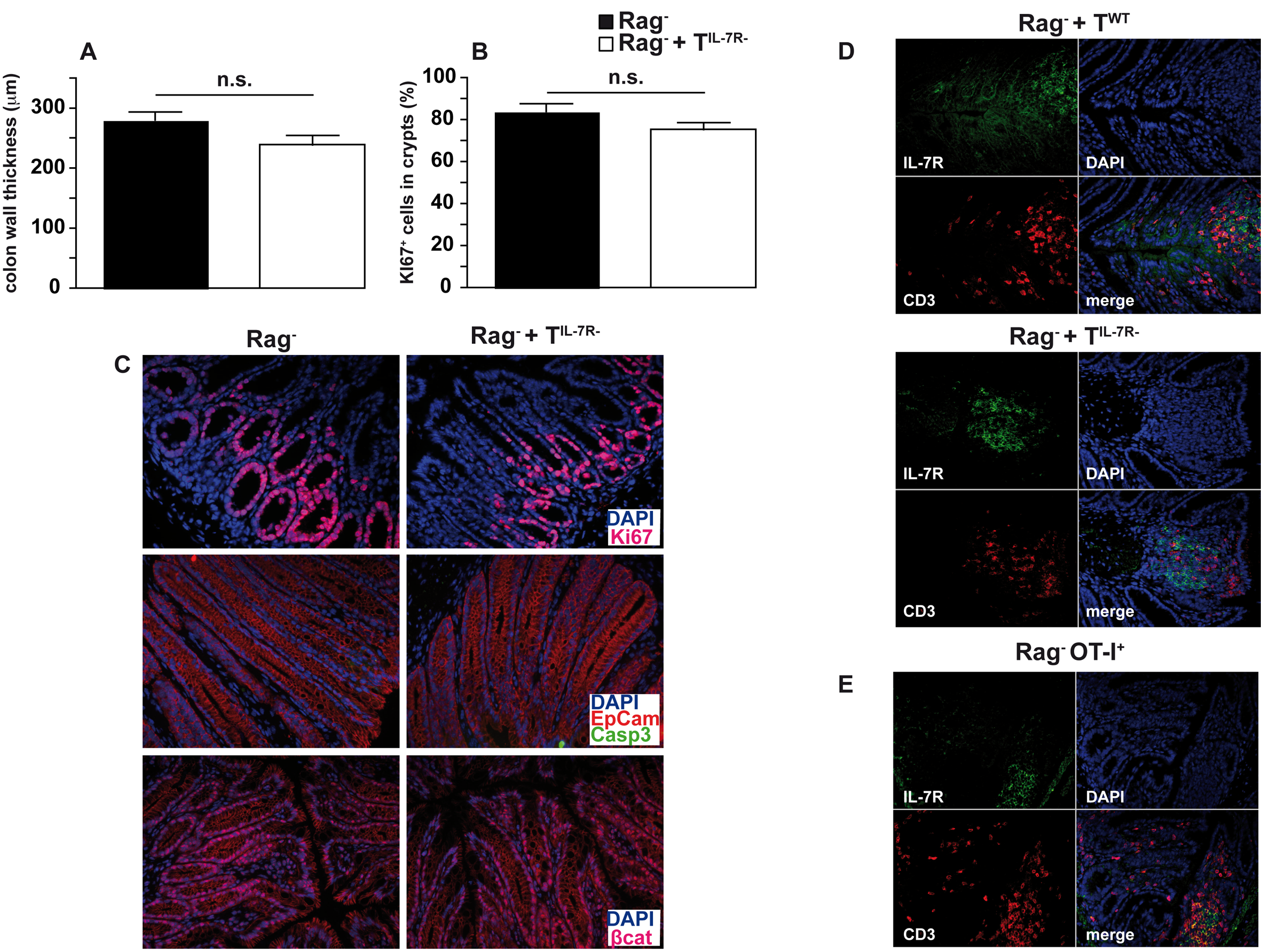

Supplement: Figure S5 — IL-7R-deficient T cells do not regulate IEC homeostasis. (A–D) Rag− mice were reconstituted with 1×10^6^ MACS-sorted CD4+ and CD8+ T lymphocytes isolated from spleens and lymph nodes of IL-7R− mice (Rag−+TIL-7R-; n = 3). Controls were left untreated (Rag−; n = 4). Colon sections were stained with DAPI and (A–C) antibodies for Ki67, EpCam, Cleaved-caspase 3 (Casp3) or β-catenin (βcat) 100 days after transfer. Data are representative for 2 independent staining reactions per mouse. Shown are (A) colon wall thickness (µm) and (B) the percentage of Ki67+ cells in crypts as mean values+SEM. Values were not significantly different (n.s.: p>0.05; Student's t test). (D) Colon sections from Rag− mice reconstituted with WT T lymphocytes (Rag−+TWT; see Figure 5) or IL-7R− T cells (Rag−+TIL-7R-) or (E) untreated Rag−OT-I+ mice were stained with DAPI and antibodies for IL-7R and CD3 to visualize transferred T cells (CD3+IL-7R+/−) and innate lymphoid cells (CD3−IL-7R+). Results are representative for 2 independent staining reactions per mouse. (TIF) [file pone.0031939.s005.tif]

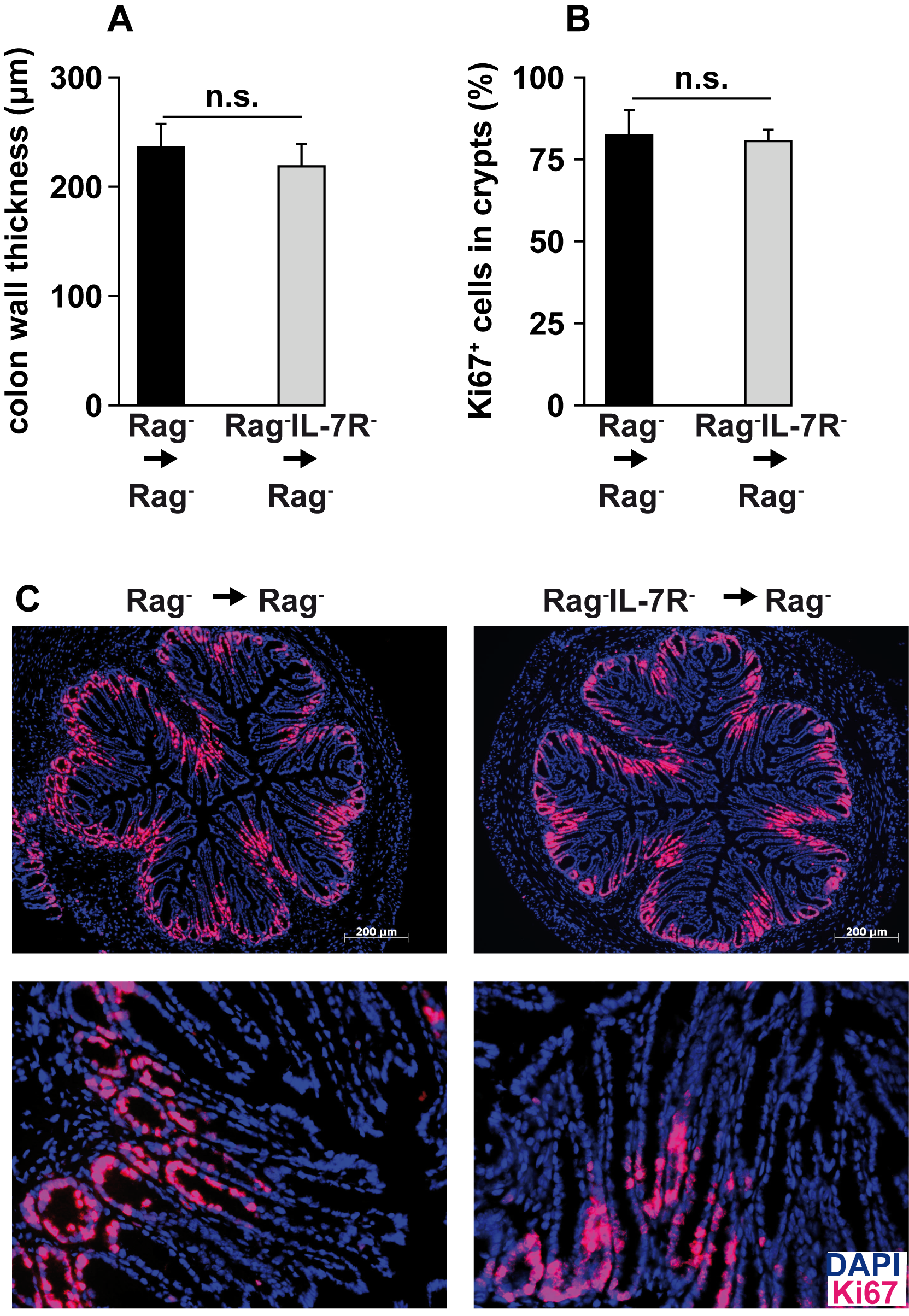

Supplement: Figure S6 — IL-7R expression on non-hematopoietic cells is sufficient for IEC hyperplasia in the colon of Rag− mice. (A–C) Lethally irradiated Rag− mice were reconstituted with bone marrow cells from Rag− mice (Rag−→Rag−; n = 4) or Rag−IL-7R− (Rag−IL-7R−→Rag−; n = 3). (A) Colon wall thickness (µm; 46–56 individual measurements per group) and (B) the percentage of Ki67+ cells in crypts (at least 500 nuclei per group) were determined 9 weeks later. (A, B) Shown are mean values+SD. Statistically significant values are indicated: n.s., not significant; * p<0.05 and ** p<0.01 (Student's t test). (D) Colon sections from Rag−→Rag− (n = 4) and Rag−IL-7R−→Rag− chimeras (n = 3) were stained with DAPI and antibodies for Ki67. Upper row: 100× magnification; lower row: 400× magnification. Data are representative 2–3 staining reactions per mouse. (TIF) [file pone.0031939.s006.tif]

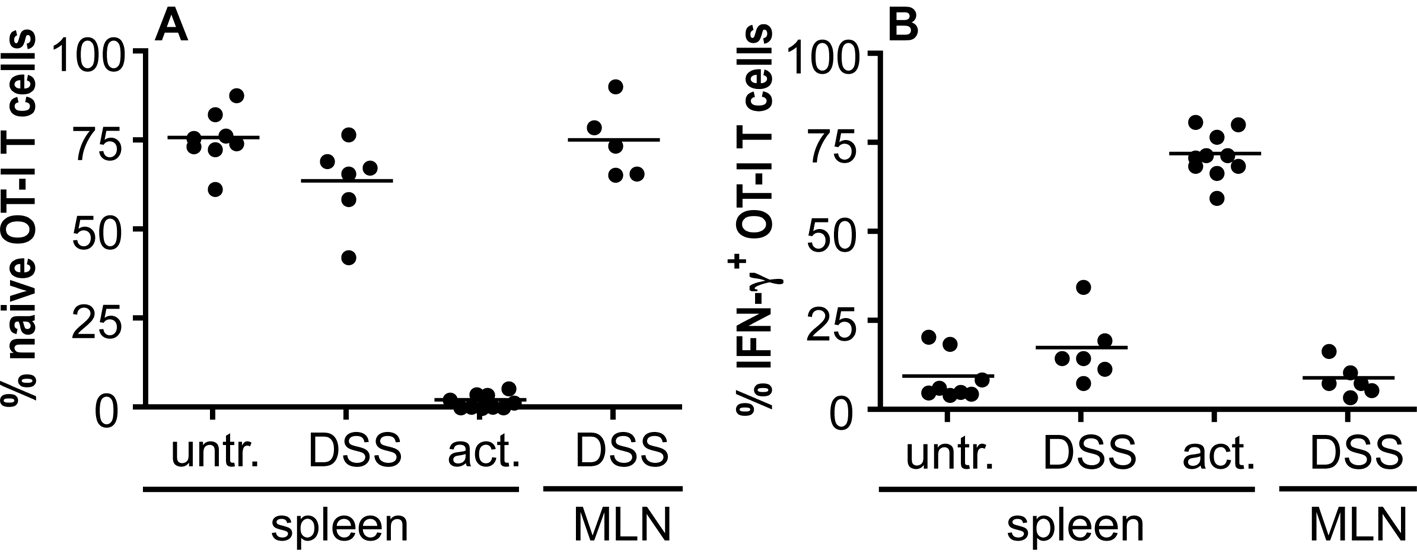

Supplement: Figure S7 — DSS treatment does not activate CD8+ OT-I T cells. CD8+ OT-I T cells were isolated from the spleen and mesenteric lymph node (MLN) of untreated (untr.; n = 8) and DSS-treated (DSS; n = 6; see Figure 6) Rag−OT-I+ mice. Activated CD8+ OT-I T cells (act.) were recovered from spleens of Rag− mice (n = 10) 21–25 days after adoptive transfer and homeostatic expansion of 1×10^6^ cells. (A) The percentage of naive CD44loCD62Lhi OT-I T cells was determined by flow cytometry after gating on CD8+Thy1.1+ cells. (B) CD8+ OT-I cells from the indicated sources were stimulated with their cognate peptide SIINFEKL for 6 hours and the percentage of Interferon-© (IFN-©)-positive cells was determined by flow cytometry after gating on CD8+Thy1.1+ cells. Data are representative for 1 (DSS) and 2 (act.) experiment(s). (TIF) [file pone.0031939.s007.tif]

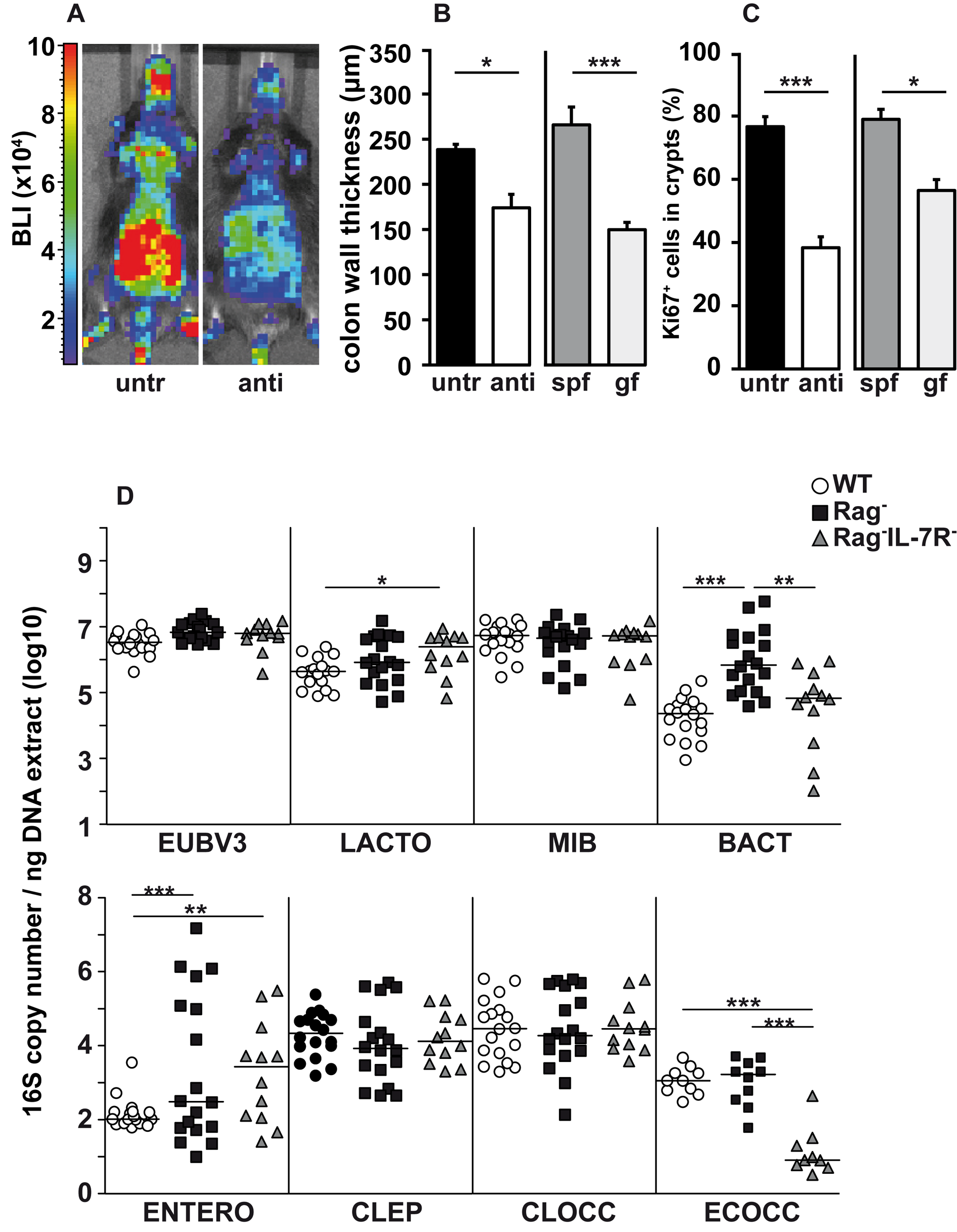

Supplement: Figure S8 — The commensal microflora promotes intestinal IL-7 production, which induces IEC hyperplasia and alterations in the commensal microflora. (A) Rag−IL-7GCDL mice (n = 6) were treated with antibiotics for 49 days as described previously [20]. The same mouse prior to (untr) and after antibiotic treatment (anti) is shown. BL is shown in photons per s per cm2 per steradian. Similar results were obtained after antibiotic treatment for 21 days in 3 additional experiments (n = 21). (B) Colon wall thickness (µm) and (C) the percentage of Ki67+ cells in crypts were determined in colon sections from untreated (untr; n = 3) and antibiotic-treated Rag−IL-7GCDL mice (anti; n = 6) as well as specific-pathogen-free (spf; n = 3) and germ-free Rag− mice (gf; n = 10). 5–7 individual crypts per mouse were analyzed. Shown are mean values+SEM. Statistically significant values are indicated: * p<0.05 and *** p<0.001 (Student's t test). (B, C) For antibiotic-treated mice, crypt length and the percentage of Ki67+ cells in crypts were analyzed for the representative experiment shown in (A). For germ-free mice, pooled data from two independent experiments are shown. Data are representative for 2–3 independent staining reactions per mouse. (D) Feces samples from WT (n = 18), Rag− (n = 18) and Rag−IL-7R− (n = 12) mice were analyzed by qRT-PCR to determine the copy number of eubacterial 16 S rDNA (EUBV3) and 16 S rRNA from Lactobacillus-group (LACTO), Mouse Intestinal Bacteroides (MIB), Bacteroides/Prevotella-group (BACT), gamma Proteobacteria/Enterobacteriaceae (ENTERO), Clostridium leptum subgroup (CLEP), Clostridium coccoides subgroup and Enterococcus (ECCOC). Shown are 16 S copy numbers per ng DNA (log10) for individual mice and the median for each experimental group. Statistically significant values are indicated: * p<0.05, ** p<0.01, *** p<0.001 (Mann-Whitney-U-Test). (TIF) [file pone.0031939.s008.tif]
