# Supplementary material for: Interleukin-7 Links T Lymphocyte and Intestinal Epithelial Cell Homeostasis
Source: PLoS One. 2012 Feb 27;7(2):e31939. doi: 10.1371/journal.pone.0031939 (PMC3288069; doi:10.1371/journal.pone.0031939)
Supplement: Information S1 — Experimental procedures for the generation of bone marrow chimeras (Figure S6) and the analysis of the intestinal microflora (Figure S8) are described. Specific references are included. (DOCX) [file pone.0031939.s009.docx]

**Supporting Information**

**Materials and Methods**

***Analysis of the intestinal microflora by real-time PCR and quantification of 16S rRNA gene sequences.*** DNA extractions from intestinal luminal contents were prepared as described previously [1]. The DNA extracts and the plasmids were quantified with Quant-iT PicoGreen reagent (Invitrogen) and were all adjusted to 1 ng DNA/µl. The abundance of specific intestinal bacterial groups was measured by quantitative real-time PCR based on a SYBR Green Assay using a LightCycler 1.5 (Roche Diagnostics) PCR with group-specific 16S rRNA gene primers (Tib Molbiol) as listed in Table 1. Each reaction mixture (20 µl) was composed of 12.2 µl water (Sigma-Aldrich, USA), 2 µl 10**×** Buffer I and 2 µl 25mM MgCl_2_ (Applied Biosystems), 0.4 µl PCR nucleotide mix (each dNTP 10 mM, Roche Diagnostics), 0.1 µl BSA (20 mg/ml, Fermentas), 0.1 µl of a 1:200 dilution of SYBR Green I (10.000**×** concentrate, Invitrogen), 0.4 µl of AmpliTaq DNA polymerase (2.5 U/µl, Applied Biosystems), 0.4 µl of each of the specific primers (30 µM, Tib MolBiol) and 2 µl DNA extract adjusted to 1 ng/µl. The amplification started with an initial denaturation at 95°C for 2 min followed by 40 cycles of 95°C for 10 s, annealing at 60°C for 10 s, and elongation at 72°C for 30 s. Fluorescence data were acquired at 80°C (75°C for MIB/BACT, 75°C for ECOCC) after each cycle. After amplification melting curves for checking the targeted PCR products were obtained by heating from 60°C to 95°C at a rate of 0.05°C/s, with continuous fluorescence collection. Data analysis was done with LightCycler Software 3.5.28 using the second derivative maximum method.

As reference for quantification standard curves with tenfold serial dilutions of plasmids (ranging from 2 × 10^8^ to 2 × 10^2^ copies) were generated for each run. The real-time PCR primers were first used to amplify cloned 16S rDNA of reference strains. Each PCR product was cloned into pCR2.1-TOPO using the TOPO-TA cloning kit (Invitrogen) following the manufacturer‘s recommendations. The number of 16S rRNA gene copies/ng DNA of each sample was determined, not the actual bacterial numbers or CFU.

| **Target** | **Reference Strain** | **Sequence (5´ to 3´)** | **Reference** |
| --- | --- | --- | --- |
| **Domain Bacteria**  (targets 16S V3 region) | Escherichia coli ATCC 25922 | F: CGGYCCAGACTCCTACGGG  R:TTACCGCGGCTGCTGGCAC | [2] |
| **Clostridium 16S rRNA cluster IV**  [*Clostridium leptum* subgroup,  includes *Faecalibacterium*  *(Fusobacterium) prausnutzii*] | Clostridium leptum DSMZ 753 | F: TTACTGGGTGTAAAGGG  R: TAGAGTGCTCTTGCGTA | [3] |
| **Clostridium 16S rRNA cluster XIVa and XIVb**  (*Clostridium coccoides –*  *Eubacterium rectale* subgroup) | Clostridium coccoides DSMZ 935 | F: AAATGACGGTACCTGACTAA  R: CTTTGAGTTTCATTCTTGCGAA | [4] |
| **Bacteroides group,** including  *Prevotella* and *Porphyromonas* | Bacteroides ovatus DSMZ 1896 | F: GAAGGTCCCCCACATTG  R: CAATCGGAGTTCTTCGTG | [5] |
| **gamma-Proteobacteria/Enterobacteriaceae** | Escherichia coli ATCC 25922 | F: AAACTCAAATGAATTGACGG  R: CTTTTGCAACCCACTCC | [6] |
| **Lactobacillus group** including  *Leuconostoc*, *Pediococcus*,  *Aerococcu*s and *Weissella* but not  *Enterococcus* or *Streptococcus* | Lactobacillus acidophilus DSM 20079 | F: CACCGCTACACATGGAG  R: AGCAGTAGGGAATCTTCCA | [7]  [8] |
| **Enterococcus genus** | Enterococcus faecalis DSM 20478 | F: CCTTATTGTTAGTTGCCATCATT  R: ACTCGTTGTACTTCCCATTGT | [9] |
| **Mouse Intestinal Bacteroides** | MIB plasmid 16-1 | F: CCAGCAGCCGCGGTAATA  R: CGCATTCCGCATACTTCTC | [10] |

***Generation of bone marrow chimeras.*** CD45.1^+^ Rag^-^ mice were irradiated with 8.5 Gy and reconstituted with 1.8-2.4 x 10^7^ BM cells from CD45.2^+^ Rag^-^IL-7R^-^ or CD45.1^+^ Rag^-^ donors. Mice were treated for 4 weeks with antibiotics (Dimitridazole 4g/l, Sulfadoxine 100mg/l, Trimethoprim 20mg/l) via the drinking water. The degree of chimerism was determined by flow cytometry in the peripheral blood 8.5 weeks post BM transfer. More than 95% of peripheral blood leukocytes of CD45.1^+^ Rag^-^ recipients were derived from the CD45.2^+^ bone marrow of donor Rag^-^IL-7R^-^ mice.

**References**

1 Heimesaat MM, Bereswill S, Fischer A, Fuchs D, Struck D, et al (2006) Gram-negative bacteria aggravate murine small intestinal Th1-type immunopathology following oral infection with Toxoplasma gondii. J Immunol 177: 8785-8795.

2 Lee DH, Zo YG, Kim SJ (1996) Nonradioactive method to study genetic profiles of natural bacterial communities by PCR-single-strand-conformation polymorphism. Appl Environ Microbiol 62: 3112-3120.

3 Van Dyke MI, McCarthy AJ (2002) Molecular biological detection and characterization of Clostridium populations in municipal landfill sites. Appl Environ Microbiol 68: 2049-2053.

4 Matsuki T, Watanabe K, Fujimoto J, Miyamoto Y, Takada T, et al (2002) Development of 16S rRNA-gene-targeted group-specific primers for the detection and identification of predominant bacteria in human feces. Appl Environ Microbiol 68: 5445-5451.

5 Bartosch S, Fite A, Macfarlane GT, McMurdo ME (2004) Characterization of bacterial communities in feces from healthy elderly volunteers and hospitalized elderly patients by using real-time PCR and effects of antibiotic treatment on the fecal microbiota. Appl Environ Microbiol 70: 3575-3581.

6 Kühbacher T, Ott SJ, Helwig U, Mimura T, Rizzello F, et al (2006) Bacterial and fungal microbiota in relation to probiotic therapy (VSL#3) in pouchitis. Gut 55: 833-841.

7 Heilig HG, Zoetendal EG, Vaughan EE, Marteau P, Akkermans AD, et al. (2002) Molecular diversity of Lactobacillus spp. and other lactic acid bacteria in the human intestine as determined by specific amplification of 16S ribosomal DNA. Appl Environ Microbiol 68: 114-123.

8 Walter J, Hertel C, Tannock GW, Lis CM, Munro K, et al. (2001) Detection of Lactobacillus, Pediococcus, Leuconostoc, and Weissella species in human feces by using group-specific PCR primers and denaturing gradient gel electrophoresis. Appl Environ Microbiol 67: 2578-2585.

9 Rinttilä T, Kassinen A, Malinen E, Krogius L, Palva A (2004) Development of an extensive set of 16S rDNA-targeted primers for quantification of pathogenic and indigenous bacteria in faecal samples by real-time PCR. J Appl Microbiol 97: 1166-1177.

10 Barman M, Unold D, Shifley K, Amir E, Hung K, et al (2008) Enteric salmonellosis disrupts the microbial ecology of the murine gastrointestinal tract. Infect Immun 76: 907-915.
